# Pathway to Polyradicals: A Planar and Fully π-Conjugated Organic Tetraradical(oid)

**DOI:** 10.1021/acs.jpclett.4c00686

**Published:** 2024-05-08

**Authors:** Sergi Betkhoshvili, Ibério de P. R. Moreira, Jordi Poater, Josep Maria Bofill

**Affiliations:** † Departament de Química Inorgànica i Orgànica and IQTCUB, Universitat de Barcelona, Martí i Franquès 1-11, 08028 Barcelona, Spain; ‡ Departament de Ciència de Materials i Química Física, Secció de Qumíca, Física and IQTCUB, Universitat de Barcelona, Martí i Franquès 1-11, 08028 Barcelona, Spain; ¶ ICREA, Pg. Lluís Companys 23, 08010 Barcelona, Spain

## Abstract

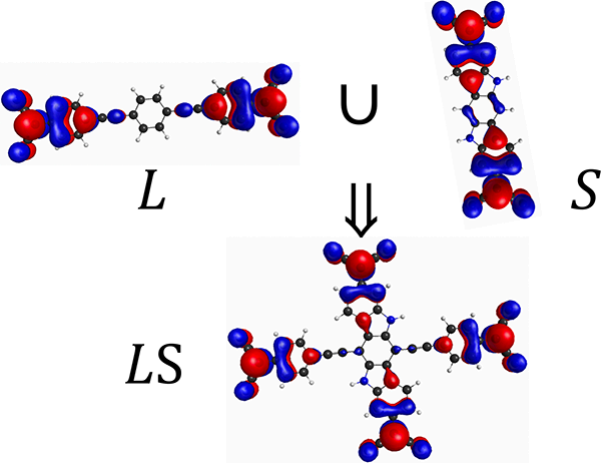

In this work, we provide a general strategy to stabilize the ground state of polyradical(oid)s and make higher spin states thermally accessible. As a proof of concept, we propose to merge two planar fully π-conjugated diradical(oid)s to obtain a planar and cross-conjugated tetraradical(oid). Using multireference quantum chemistry methods, we show that the designed tetraradical(oid) is stabilized by aromaticity and delozalization in the π-system and has six thermally accessible spin states within 1.72 kcal/mol. Analysis of the electronic structure of these six states of the tetraradical(oid) shows that its frontier π-system consists of two weakly interacting subsystems: aromatic cycles and four unpaired electrons. Conjugation between unpaired electrons, which favors closed-shell structures, is mitigated by delocalization and the aromaticity of the bridging groups, leading to the synergistic cross-coupling between two diradical(oid) subunits to stabilize the tetraradical(oid) electronic structure.

Purely organic magnetic materials are emerging as promising candidates to substitute scarce and environmentally harmful transition metal and lanthanide compounds for many applications in organic electronics. This new generation of organic materials shows magnetic properties with potential applications in flexible organic electronics,^1,2^ photonics,^2−5^ spintronics,^6−8^ and organic quantum devices.^9^ The magnetic properties of these materials arise from molecular entities (isolated molecules or defined groups in a polymeric structure) with unpaired electrons, usually called radical centers. The synthesis of stable polyradicals with large spin angular momentum is still a major challenge due to their high chemical reactivity. The stability of the radical centers can be enhanced by surrounding bulky groups such as in the sterically hindered triphenylmethyl radical^10^ or nitronyl/nitroxide polyradicals.^11−13^ However, this strategy usually leads to difficulties in connecting radical centers close enough to form small polyradicals. Another strategy for the stabilization of unpaired electrons is through delocalization via π-conjugation in planar molecular structures.^1,8,13−22^ This approach is effective to design systems with any number of unpaired electrons and can lead to singlet or doublet ground states with thermally accessible high-spin states (polyradicals) or singlet or doublet ground states with a little higher-lying high-spin states (polyradicaloids). The most widely studied polyradical(oid)s are diradical(oid)s with potential applications in organic electronics and spintronics, n-channel or ambipolar field effect transistors (FETs), organic magnetic materials, molecular switches, singlet fission with solar energy conversion capability, batteries, nonlinear optics, functional dyes, and photodynamic therapy.^1,6,23−37^ The history of higher-order polyradicals starts in 1964 with the first synthesized triradical^38^ and tetraradical,^39^ after which many experimental and theoretical studies followed.^11−21,40−55^

When discussing polyradicals, we must distinguish between two extremes: the first is a polymer of monoradicals, which can be a chain of repeating radical units, and the second is a single molecule with several unpaired electrons. Our focus of this work is to devise a strategy to generate single molecules with several unpaired electrons. Motivated by the difficulties in stabilizing high spin states in organic molecules, in this Letter, we present a new and general strategy of designing polyradical(oid) molecules with thermally accessible (i.e., within 2 kcal/mol from the ground state) spin states. Through a suitable unification of two fully π-conjugated and planar diradical(oid)s, we propose a new cross-conjugated tetraradical(oid) with six thermally accessible spin states of two singlets, three triplets and one quintet. This strategy envisages generating more complex planar π-conjugated polyradical(oid)s. Since we cannot be experimentally certain that a quintet state is thermally accessible, we use the term “tetraradical(oid)” to express generality.

The tetraradical(oid) we designed is inspired from the diradical(oid)s that are either already synthesized or resemble the structures of well-known diradical(oid)s. The diradical(oid) 2,2′-(5,11-dihydroindolo[3,2-*b*]carbazole-3,9-diyl)dimalononitrile (***S***) shown in Figure 1a has been synthesized and computationally studied. Its trivial derivative is also a diradical(oid) that dimerizes readily, proving its significant diradical character.^56,57^ Since diradical(oid) ***S*** has its substituteable hydrogens in *para* positions from one another in the central benzene ring, we could substitute both of them with radicals to obtain a tetraradical(oid). It is more pertinent to complete the building of the tetraradical(oid) from ***S*** by merging it with structures that are stable diradical(oid)s. Such known diradical(oid)s are homologous Thiele’s (*n* = 1), Chichibabin’s (*n* = 2) or Müller’s (*n* = 3) hydrocarbons with structure Ph_2_C=(Ph)_*n*_=CPh_2_, with the latter having the highest diradical character.^58−60^ If we insert acetylene residues between benzene rings of Müller’s hydrocarbon, we avoid the steric hindrance without changing the topology of the π-conjugation. Furthermore, since ***S*** has cyano terminal groups and it is still a diradical(oid), we could substitute terminal phenyl groups of the Müller’s hydrocarbon with cyano groups. Upon such changes, we obtain 2,2′-((1,4-phenylenebis(ethyne-2,1-diyl))bis(4,1-phenylene))dimalononitrile (***L***) shown in Figure 1b, which represents the additional diradical(oid) component toward building a tetraradical(oid) ***LS***. As shown in Figure 1, for both diradical(oid)s ***S*** and ***L***, Ovchinnikov’s rule^61^ predicts a singlet open-shell ground state (G.S.), as the antiferromagnetic coupling is transmitted via an antiferromagnetic coupling unit (ACU).^24^ For ***S*** this is corroborated by experiments, and for ***L*** this could be extrapolated from Müller’s hydrocarbon, which has a singlet G.S. Ovchinnikov’s rule also predicts that the system obtained from the (approximate) conceptual union of ***L*** and ***S***, the tetraradical(oid) 2,2′-(6,12-bis((4-(dicyanomethyl)phenyl)ethynyl)-5,11-dihydroindolo[3,2-*b*]carbazole-3,9-diyl)dimalononitrile (***LS***) given in Figure 2, should also have a singlet open-shell G.S., as verified in this Letter.

**Figure 1:**
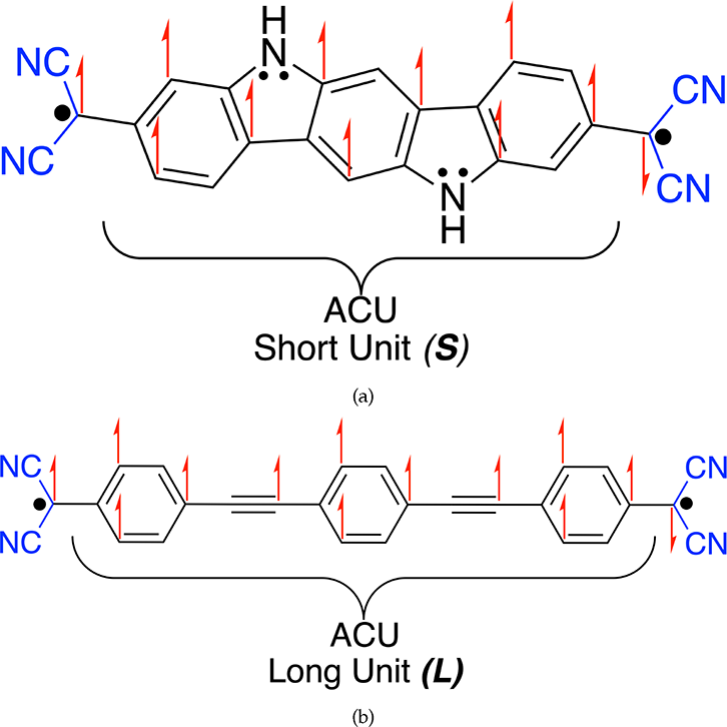
Demonstration of Ovchinnikov’s rule with antiferromagnetic coupling units (ACUs) to determine the ground state of each diradical(oid) substructure (a) ***S*** and (b) ***L*** that conceptually makes up the tetraradical(oid) ***LS***.

**Figure 2:**
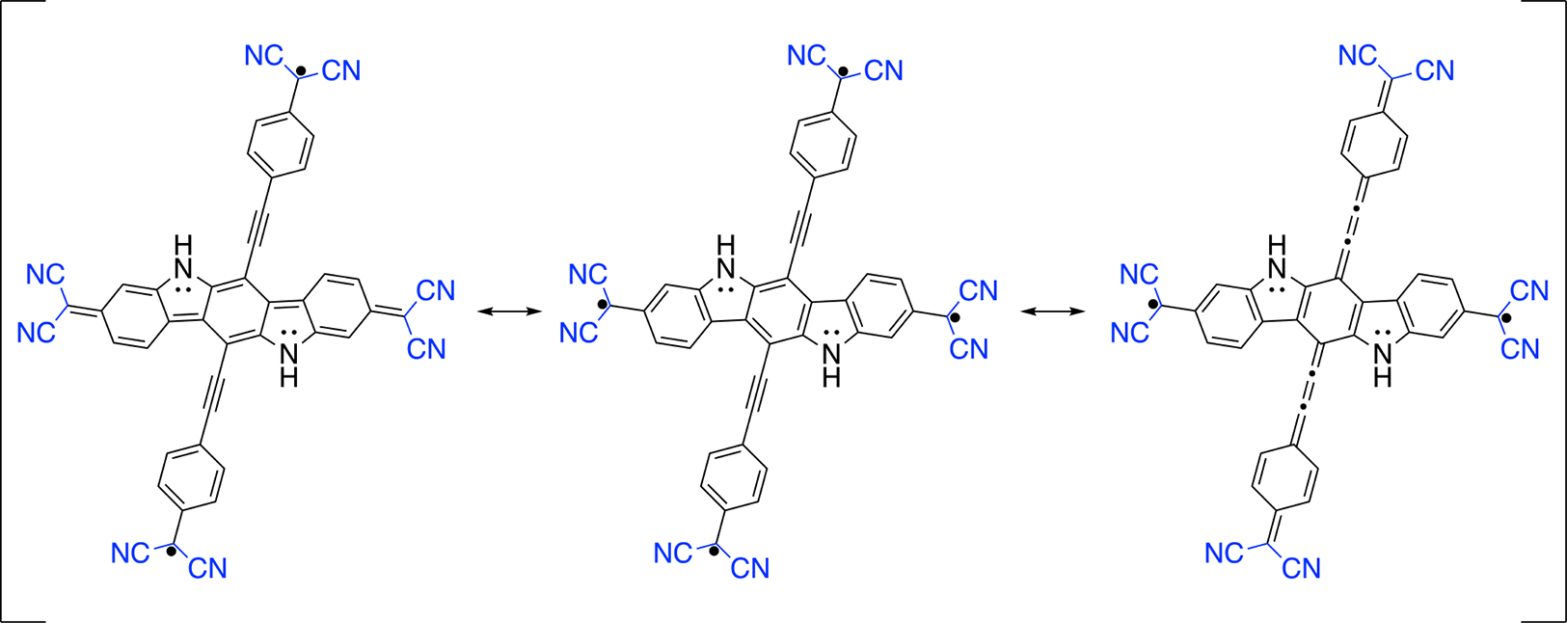
Resonance structures of the 2,2′-(6,12-bis((4-(dicyanomethyl)phenyl)ethynyl)-5,11-dihydroindolo[3,2-*b*]carbazole-3,9-diyl)dimalononitrile ***LS***. On the left, ***S*** is quinoidal and ***L*** diradical; this resonance structure is referred to as ***LDSQ***. In the middle, both ***S*** and ***L*** are diradical, thus referred as ***LDSD***. On the right, ***S*** is diradical and ***L*** is quinoidal, thus referred as ***LQSD***.

Since polyradical species are very difficult to isolate, quantum chemical calculations are important tools for studying and analyzing their electronic structure properties. The geometry for each of the structures, ***L***, ***S*** and ***LS***, was optimized with the Slater-type all-electron triple-ζ basis set (TZP)^62^ with the BLYP exchange-correlation functional^63,64^ within unrestricted Kohn–Sham (UKS) density functional theory (DFT).^65^ It must be remarked that a planar structure with *C*_2*h*_ symmetry results for ***LS*** whether the starting geometry is planar or not (refer to section S2.1 in the Supporting Information for details). After geometry optimization, ***LS*** was tested by broken-symmetry unrestricted Hartree–Fock (UHF) and found to be a possible tetraradical(oid) (see section S2.3 and Figure S3 in the Supporting Information). Moreover, single-point energy UKS DFT benchmark with hybrids of PBE^66,67^ showed UKS DFT was insufficient for the description of all spin states of ***LS*** (section S4 in the Supporting Information).

For an appropriate description of an electronic structure of a tetraradical(oid), a qualitatively correct wave function can be obtained by multiconfigurational self-consistent field (MCSCF) methods such as complete active space SCF (CASSCF), also known as full optimized reaction space (FORS),^68−75^ which describes the so-called nondynamic correlation. CASSCF calculations were run from UHF natural orbitals (see below) guess as they are one of the best guess orbitals for CASSCF calculations.^76^ In order to explore the wave function of the ***LS*** system in greater detail, CAS configuration interaction (CASCI) calculations with (4,4) and (16,16) active spaces were performed (see section S2.4 in the Supporting Information for details). All calculations other than geometry optimizations were done with Dunning’s correlation-consistent double-ζ basis set cc-pVDZ.^77^

According to CASSCF(14,14) and CASCI(14,14) or CASCI(16,16) calculations,^78^***L*** has greater diradical character than ***S***, and CASSCF(14,14) calculations show that both have a singlet open-shell ground state with singlet–triplet gaps (Δ*E*_*S*–*T*_) of 1.63 kcal/mol for ***S*** and 0.11 kcal/mol for ***L*** (section S7 in the Supporting Information).

Referring to diradical(oid)s as ***S*** and ***L*** translates into referring to singly occupied natural orbitals (SONOs) given in Figure 3 for the description of the spin states of ***LS*** given in Table1. Natural orbitals (NOs) are eigenvectors of first-order density matrix operators, and a corresponding eigenvalue to the particular NO is its occupation number^79^ (denoted as *n*_*NO*_). The highest occupied NO (HONO) is defined as the orbital that has the lowest occupation number *n*_*NO*_ among NOs with *n*_*NO*_ ≥ 1. The lowest unoccupied NO (LUNO) is defined as the orbital that has the highest *n*_*NO*_ among NOs with *n*_*NO*_ ≤ 1.

**Figure 3:**
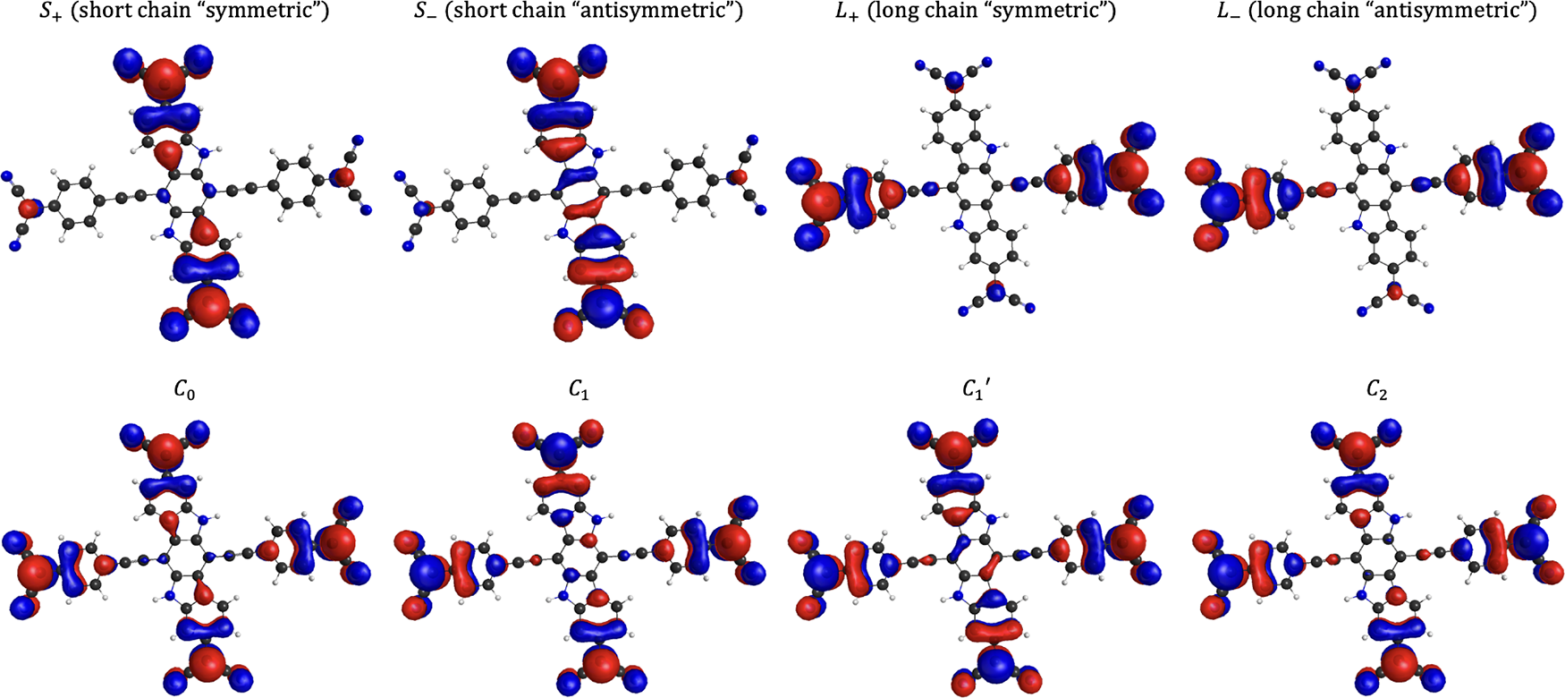
Symbolic assignations to singly occupied natural orbitals that appear for frontier orbitals in the CASSCF and CASCI solutions. Orbitals are shown as isosurfaces with a value of 0.015.

**Table 1:** CASSCF(16,16) Results^a^ with Energy Gaps from the Ground State (G.S.)^b^

state	symmetry	HONO – 1		HONO		LUNO		LUNO + 1		Δ*E* from G.S. (cm^–1^)
*S*_0_	*A*_*g*_	*S*_+_	1.221	*L*_+_	1.043	*L*_–_	0.955	*S*_–_	0.781	0.00
*T*_0_	*B*_*u*_	*S*_+_	1.222	*L*_–_	1.000	*L*_+_	0.998	*S*_–_	0.780	56.76
*T*_1_	*B*_*u*_	*L*_+_	1.058	*S*_+_	0.997	*S*_–_	0.995	*L*_–_	0.948	532.93
*S*_1_	*A*_*g*_	*C*_0_	1.059	*C*_1_	1.011	*C*_1_^*′*^	0.977	*C*_2_	0.951	534.77
*T*_2_	*A*_*g*_	*C*_0_	1.043	*C*_1_	1.021	*C*_1_^*′*^	0.975	*C*_2_	0.959	549.13
*Q*_0_	*A*_*g*_	*C*_0_	1.002	*C*_2_	0.999	*L*_–_	0.999	*S*_–_	0.997	602.74

a Detailed data for CASSCF are given in Tables S1–S4 in the Supporting Information for CASSCF(4,4), CASSCF(10,10), CASSCF(14,14), and CASSCF(16,16), respectively.

b Under columns HONO–LUNO are CASSCF NO identities from Figure 3 and their occupation numbers.

Remarkable qualitative consistency is observed throughout CASSCF(4,4), (10,10), (14,14), and (16,16) results (Tables S1–S4 in section S3.1 of the Supporting Information). CASSCF description of ***LS*** for every active space size shows four nearly singly occupied NOs for six spin states in the low-energy spectrum. Thus, ***LS*** is indeed a tetraradical(oid). Comparing the extremes, CASSCF(4,4) results given in Table S1 (Supporting Information) and CASSCF(16,16) results given in Table 1 show that we have six spin states of ***LS*** within 283.41 and 602.74 cm^–1^ (0.810 and 1.723 kcal/mol), respectively. Both sets of calculations show qualitatively the same picture for each state, as demonstrated by inspecting Table S1 (Supporting Information) and Table 1 with orbitals notations given in Figure 3. Notable differences are that energy gaps from ground state for each state are about twice as much for CASSCF(16,16) as for CASSCF(4,4) and the ordering between *S*_1_ and *T*_1_ is switched. However, the energy gap between *S*_1_ and *T*_1_ is 2.77 cm^–1^ (0.0079 kcal/mol) for CASSCF(4,4) and 1.84 cm^–1^ (0.0053 kcal/mol) for CASSCF(16,16), which are much smaller than the error scale of the employed method. Overall, the CASSCF method unequivocally points to the high tetraradical character of ***LS***.

For planar polyradicals, interactions between spin centers can be approximately characterized by the Heisenberg–Dirac–van Vleck Hamiltonian (*Ĥ*_*HDVV*_ = −∑_*i*<*j*_*J*_*ij*_**Ŝ**_*i*_**·Ŝ**_*j*_),^80−83^ which describes open-shell systems as particle-per-site model of spin centers. Moreover, one must also build an effective Hamiltonian^84,85^ for the system and correspond it to *Ĥ*_*HDVV*_ to obtain exchange-coupling constants. CASCI(16,16) results run with a neutral determinant basis of *S*_*z*_ = 0 subspace showed that the exchange-coupling constant (*J*) for radical centers in subsystem ***S*** is *J*_*S*_ = 312.06 cm^–1^ (positive value means an antiferromagnetic interaction), while in subsystem ***L*** it is *J*_*L*_ = 43.34 cm^–1^ (see Tables S7 and S8 in section S3.1 and section S3.2 in the Supporting Information for details and theoretical background). Furthermore, there is quite a strong coupling between the radical center of ***S*** with the radical center of ***L*** on the opposite side of indole nitrogen with value *J*_*a*_ = 59.92 cm^–1^ and a weaker coupling between the radical center of ***S*** with the radical center of ***L*** on the same side of the indole nitrogen with value *J*_*b*_ = 12.92 cm^–1^. We note that the reason *J*_*a*_ is much greater than *J*_*b*_ is because *J*_*a*_ is a coupling between spin centers arising from the unpaired electrons that are conjugated directly so that they *could* pair up to close the shell, while electrons giving rise to spin centers coupled via *J*_*b*_ could not (see ***LRSR*** and ***LRSR′*** resonance structures of ***LS*** in Figure S1 of the Supporting Information). *J*_*a*_ is the interaction that most reflects the *synergy* between ***S*** and ***L*** to form tetraradical(oid) ***LS***. The relative strengths of exchange-coupling constants are given in Figure 4a, and the energy spectrum of its spin states is given in Figure 4b. Upon inspection of results in Tables S1–S8 in section S3.1 of the Supporting Information, we see that CASCI(4,4) and CASCI(16,16) calculations parallel each other in terms of the determinantal build of the wave function and energy ordering of spin states and show remarkable qualitative consistency with CASSCF results. With CASCI(16,16), we find that in the description of the wave function of ***LS*** the aromaticity of the molecule^86,87^ is important (see Table S8 in section S3.1 of the Supporting Information). Hence, it can be claimed that the frontier π-electronic system of ***LS*** consists of two subsystems. The first of these is aromatic π-subspace, which is mostly delocalized within an indolo[3,2-*b*]carbazole aromatic moiety and two phenyl rings. The second of these subsystems is a set of four unpaired electrons that form the tetraradical(oid) electronic structure. These subsystems interact weakly but substantially enough to affect the energy spectrum of spin states.

**Figure 4:**
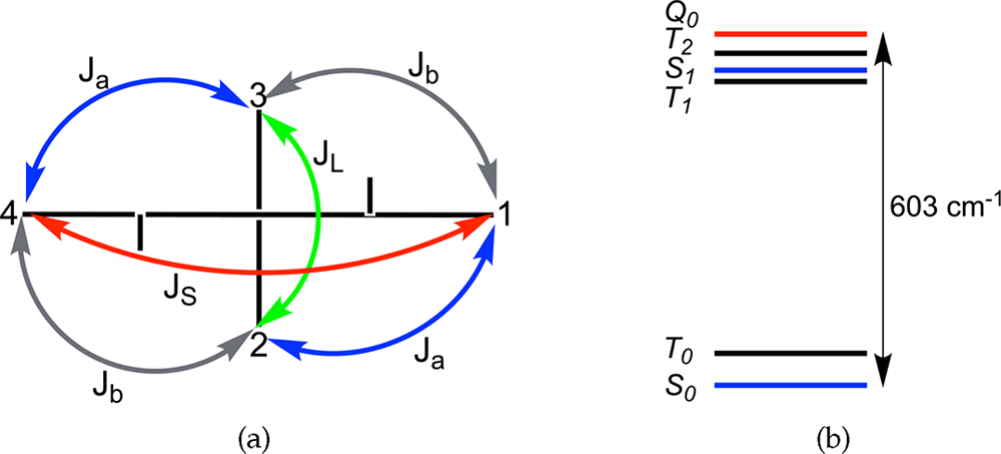
(a) Scheme of the strength of exchange-coupling interactions in the tetraradical(oid) ***LS***. Red is the strongest, blue is the second strongest, closely followed by green, and the weakest is gray. Rods sticking out from the horizontal line (1–4) correspond to the indole nitrogens. (b) Low-energy sectrum of the tetraradical(oid) ***LS***.

The possible reasons for the stabilization of this tetraradical(oid) electronic structure are symmetry, a high degree of conjugation and delocalization, the presence of bridging groups between radical centers involving *locally* aromatic benzenoid rings (Clar’s π-sextets), and the potential for so-called “*global aromaticity*” of the indolo[3,2-*b*]carbazole backbone. Upon investigation of the anisotropy of induced current density (ACID) of singlet and quintet states, which describes the response of electron currents on a perpendicular magnetic field that leads to flow of electron current around the aromatic rings,^88^ we detected continuous electron flow around the perimeter of the indolo[3,2-*b*]carbazole backbone and two phenyl rings bonded to the central benzene ring of the indolo[3,2-*b*]carbazole as given in Figure 5 for a singlet state, which is almost indistinguishable from a quintet state in this aspect (Figure S8 in section S5 of the Supporting Information). Hence, in addition to evidence from CASSCF and CASCI calculations, we have other *demonstrations* for the aromatic stabilization of this tetraradical(oid). These results are also corroborated by another measure of aromaticity, the multicenter index (MCI).^89,90^ MCI values show that benzenoid rings within ***LS***, for which results are given in Table S10 of the Supporting Information, are indeed aromatic. To explain the maintenance of open shells, we should also note that diradical resonance structures possess three more Clar’s aromatic π-sextets^91,92^ than closed-shell quinoidal structures for ***L*** (Figure S12 in the Supporting Information) and ***S*** (Figure S13 in the Supporting Information), which stabilizes the diradical form over the quinoidal form. This is also maintained in the tetraradical(oid) ***LS***, for which the resonance structure (from Figure 2) ***LDSD*** has three more Clar’s aromatic π-sextets than the resonance forms ***LDSQ*** and ***LQSD*** (and ***LRSR***/***LRSR′*** structures given in Figure S1 of the Supporting Information). Notably, subunits ***S*** and ***L*** based on *n*_*NO*_ of their frontier NOs maintain their respective diradical(oid) structure quite closely in the tetraradical(oid) ***LS***. For demonstration, we can compare *S*_0_ states from CASSCF(14,14) for ***S***, ***L***, and ***LS*** (Tables S17, S20, and S3, respectively, in the Supporting Information). Occupation numbers in the *S*_0_ state change from *n*_*HONO*_ = 1.047–1.051 and *n*_*LUNO*_ = 0.958–0.947 for ***L***, while occupation numbers in the *S*_0_ state change from *n*_*HONO*_ = 1.205–1.192 and *n*_*LUNO*_ = 0.801–0.813 for ***S***. If we view this tetraradical(oid) ***LS*** from the modular perspective of ***L*** and ***S***, it would not necessarily (and usually) be expected to combine these diradical(oid) structures in some way and still maintain electrons unpaired to form tetraradical(oid), which is usually successively more difficult as the number of unpaired electrons increases. Nonetheless, this is what we observed for ***LS***. Hence, there is a *synergy* between ***L*** and ***S*** substructures within ***LS*** that maintains all the open shells and achieves the diradical(oid) + diradical(oid) + coupling = tetraradical(oid) structure, as manifested by *J*_*a*_ = 59.92 cm^–1^. Evidently, the conceptual union of these two diradical(oid)s produces tetraradical(oid) ***LS***, which is indeed one of the smallest planar and fully π-conjugated organic tetraradical(oid)s that has been described in the scientific literature to date. Our work predicts that ***LS*** is a genuine tetraradical with six thermally accessible spin states in the low-energy spectrum, which can be experimentally validated by electron paramagnetic resonance (EPR) spectroscopy.

**Figure 5:**
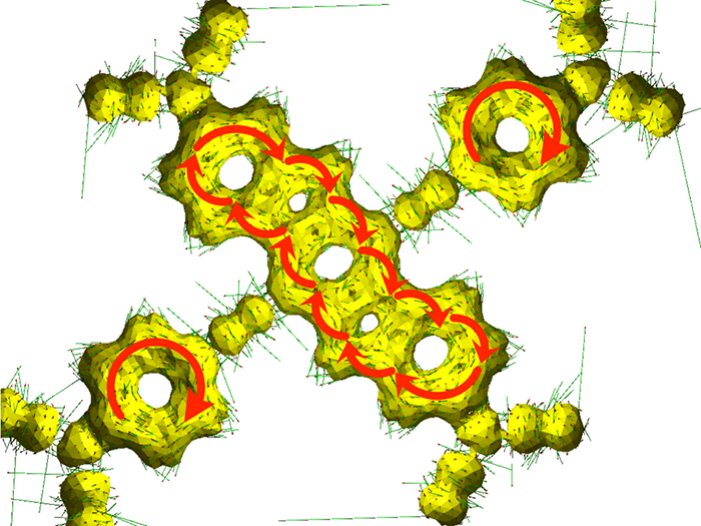
An ACID plot for a singlet state of ***LS*** calculated with the PBE0/cc-pVDZ level of theory. See the full version of the ACID plot in Figure S8a in the Supporting Information. Isosurface with value of 0.030.

Our general approach can be summarized in the following way: (1) If the polyradical is built from merging diradicals or other polyradicals, one must ensure that π-conjugation is maintained throughout the resulting structure. (2) Within a given polyradical, each diradical subunit must have an aromatic stabilization with at least two or (more favorably) three benzene rings. (3) Aromatic rings can be shared between different subunits, similarly as the central benzene ring is shared between ***L*** and ***S*** subunits within tetraradical(oid) ***LS***. (4) Upon the design of a polyradical, one can take advantage of cross-conjugation to restrict the lower bound of polyradical character, as we did to restrict the minimal polyradical character of tetraradical(oid) ***LS*** to 2.

To conclude, we provide a general strategy to design polyradicals with thermally accessible high spin electronic states. Thus, 2,2’-(6,12-bis((4-(dicyanomethyl)phenyl)ethynyl)-5,11-dihydroindolo[3,2-*b*]carbazole-3,9-diyl)dimalononitrile (***LS***) is built with this approach as a merger of ***L*** and ***S*** diradical(oid) constituents. Diradical(oid)s are merged in a way that retains π-conjugation and allows for inner electron delocalization, which is a source of stabilization. Such a mechanism can be used to stabilize hexaradicals, octadecaradicals, and higher polyradicals. Hence, this frontier toward higher polyradical(oid)s is being actively explored further by our group.

## Supporting Information Available

Optimized geometries, computational details of geometry optimization, methodology of the open-shell character test, computational details of CASSCF and CASCI, details of the model and effective Hamiltonian, results of CASSCF and CASCI calculations, results from the broken-symmetry DFT and HF benchmark, results from ACID and MCI, and study of constituent diradical(oid)s (PDF)
Transparent Peer Review report available (PDF)
