# Supplementary material for: Pathway to Polyradicals: A Planar and Fully π-Conjugated Organic Tetraradical(oid)
Source: J Phys Chem Lett. 2024 May 8;15(19):5243–9. doi: 10.1021/acs.jpclett.4c00686 (PMC11103692; doi:10.1021/acs.jpclett.4c00686)
Supplement: Supplementary file 1 — jz4c00686_si_001.pdf [file jz4c00686_si_001.pdf]

Supporting Information

Pathway to Polyradicals: A Planar and Fully π-Conjugated Organic Tetraradical(oid)

*Sergi Betkhoshvili, Ibério de P. R. Moreira, Jordi Poater, and Josep Maria Bofill**

Contents

S1. General view of the investigated tetraradical(oid) S1

S2. Computational Details S2
S2.1. Geometry optimization S2
S2.2. Remarks on the methodology S3
S2.3. Check of the qualitative electronic structure of the ground state with HF S4
S2.4. CASSCF and CASCI calculation details S4
S2.5. Broken-symmetry calculations with HF and DFT S5

S3. Calculated results S5
S3.1. CASSCF and CASCI results S5
S3.2. Results from Heisenberg-Dirac-van Vleck model and effective Hamiltonian theory S8

S4. Broken-symmetry UKS DFT and UHF benchmark results S11

S5. Study of Aromaticity of Tetraradical(oid) *LS* S12

S6. Study of Tetraradical(oid) *LSH* S15

S7. Study of Diradical(oid)s *L* and *S* S18

S8. Optimized Geometries S20

S1. General view of the investigated tetraradical(oid)

Since tetraradicals can be seen at least conceptually as two diradicals, in the present case the diradical(oid) 2,2′-(5,11-dihydroindolo[3,2-*b*]carbazole-3,9-diyl)dimalononitrile ***S*** is conceptually merged through the central benzene ring with the diradical(oid) 2,2′-((1,4-phenylenebis(ethyne-2,1-diyl))bis(4,1-phenylene))dimalononitrile ***L***, resulting in the potential tetraradical(oid) 2,2′-(6,12-bis((4-(dicyanomethyl)phenyl)ethynyl)-5,11-dihydroindolo[3,2-*b*]-carbazole-3,9-diyl)dimalononitrile ***LS***. Thus, we refer the tetraradical(oid) described in this work as ***LS***. The notations can be understood from the Figure S1 as follows: ***L*** represents **longer** chain of the molecule including the central benzenoid ring and two benzenoid ring connected to the central benzenoid ring, delimited by ethynyl residue, while ***S*** represents the **shorter** perpendicular chain in the molecule, composed of the indolocarbazole with dicyanomethylene substituents at the ends. Therefore, this notation will be used to help describe electronic structure and density distributions. Also, this notation can be adapted to describe 4 different singly occupied natural orbitals symbolically. There are five resonance structures given in Figure S1. In the top row, on the left, short (***S***) chain is quinoidal and long chain (***L***) diradical, this resonance structure will be referred as ***LDSQ***. In the , both systems short (***S***) and long (***L***) chain are diradical in the combined system ***LS***, thus, this resonance structure will be referred as ***LDSD***. On the right, the short system (***S***) is diradical and long system (***L***) is quinoidal, thus, this resonance structure will be referred as ***LQSD***. In the bottom row resonance structures, both subunits ***L*** and ***S*** are radical, hence, we refer structure on the left as ***LRSR*** and structure on the right as ***LRSR′***. In the main text of the manuscript, ***LRSR*** and ***LRSR′*** resonance structures are omitted for conciseness. Throughout this work, all orbitals are visualized as isosurfaces with value of 0.015 unless explicitly stated otherwise.

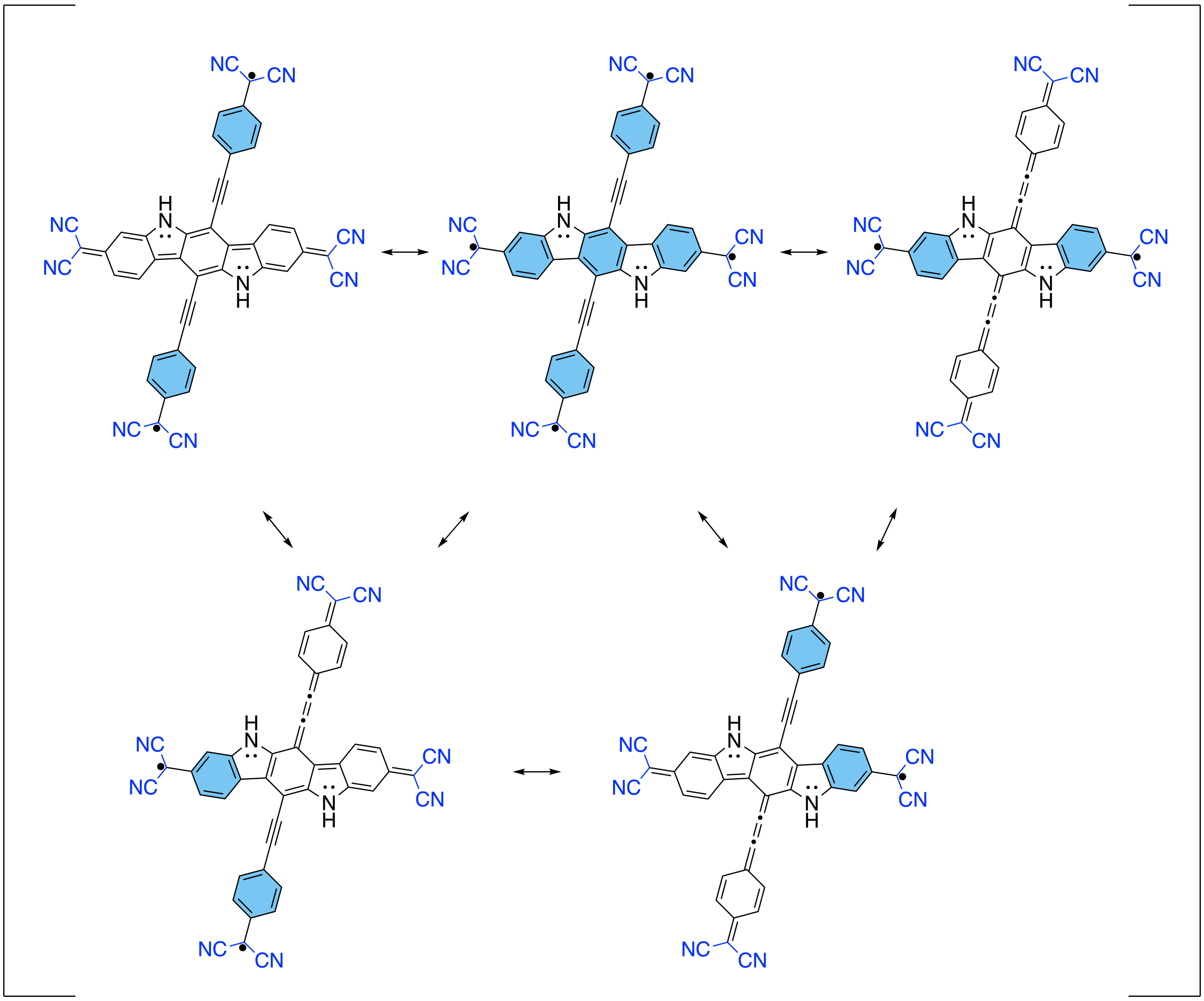

Figure S1. Resonance structures of 2,2′-(6,12-bis((4-(dicyanomethyl)phenyl)ethynyl)-5,11-dihydroindolo[3,2-*b*]carbazole-3,9-diyl)dimalononitrile ***LS***. 2,2′-(5,11-dihydroindolo[3,2-*b*]carbazole-3,9-diyl)dimalononitrile (in the figure, horizontal chain) will be referred to as ***S*** and 2,2′-((1,4-phenylenebis(ethyne-2,1-diyl))bis(4,1-phenylene))dimalononitrile (in the figure, vertical chain) will be referred to as ***L***. In the top row, leftmost resonance structure is referred to as ***LDSQ***, middle resonance structure as ***LDSD***, and rightmost resonance structure as ***LQSD***. In the bottom row, structure on the left will be referred to as ***LRSR*** and structure on the right as ***LRSR***′. Rings with Clar's aromatic π-sextet are filled-in blue.

S2. Computational Details

S2.1. Geometry optimization

For geometry optimizations of the molecules, Density Functional Theory (DFT) was used with Amsterdam Density Functional (ADF) module engine[1] within Amsterdam Modeling Suite (AMS) release of 2021 (ADF 2021.104 version). Since the molecule presented in this manuscript had been determined to possess significant tetraradical character in the preliminary tests, the unrestricted Kohn-Sham (UKS) DFT formalism with quintet state (keyword line "SpinPolarization 4" in ADF engine) was used for the geometry optimization with Generalized Gradient Approximation (GGA) exchange-correlation functional BLYP[2,3] without any relativistic corrections due to absence of heavier atoms than the first row (H, C and N). The Slater-type all-electron triple zeta (TZP)[4] basis set available in ADF was used. The self-consistent field (SCF) convergence criteria were chosen as follows: the norm of the commutator between Fock and Density matrices ([F,P]) set to $1.0 \cdot 10^{-5}$ and maximum element of the [F,P] set to $1.0 \cdot 10^{-6}$ (defaults). Numerical quality for Becke's integration grid[5,6] was set to "VeryGood", which means Lebedev angular grid order varying in range [13, 41] and number of radial points varying in range [64, 72]. Also, the numerical quality for density fitting approximation was set to "VeryGood", which means number of interpolation points varying in range [120,160] and maximum number of L-expansion varying in range [9,10]. All the other technical parameters were left as default for ADF software package. In order to verify that the optimized geometry corresponded to the minimum on the Potential Energy Surface (PES), a Hessian with respect

to nuclear coordinates was computed which turned out to have all eigenvalues positive, translating into all real calculated infrared absorption frequencies corresponding to the determined normal modes.[7] It must be mentioned that the planar structure of the molecule ***LS*** results irrespective of the planarity of the starting geometry. For example, if in the starting geometry the angle between planes defining the indolo[3,2-*b*]carbazole backbone and the plane defined by the 2-(4-dicyanomethylene-phenyl)-ethynyl substituent moieties is about 45°and 30°(for the first and the second moiety, respectively), the optimized geometry is still planar. Furthermore, even if these angles are 65°and 50°, the resulting optimized geometry is still planar. Thus, the geometry used throughout the electronic-structure calculations is an optimized structure obtained from starting planar geometry belonging to the C_{2h} point group in order to take advantage of the molecular symmetry in the downstream more expensive *ab-initio* calculations (from Figure S1, the assignation of the point group is clear for the ***LS*** system). Finally, we remark that all computations were carried out for a single, isolated molecule in the gas phase.

S2.2. Remarks on the methodology

Investigation of the electronic structure has been done with wavefunction methods rather than DFT because, as it will be shown in Figure S7 in the Section S4, DFT shows heavy functional dependence on energy spectrum of the allowed spin states. Hartree-Fock (HF) with its restricted and unrestricted formalism (RHF and UHF, respectively) is a computationally cheap but definitive method to determine whether or not the electronic structure of the molecule in the ground state is open-shell. To achieve this, all that is required is to compute RHF and UHF solutions separately and compare results. If UHF solution has lower energy than RHF solution (it can have the same energy or lower only), it means that closed-shell electronic wavefunction is unstable and the ground-state wavefunction is better described by a UHF, an open-shell, solution. Nonetheless, monoconfigurational HF and DFT methods cannot give us an appropriate description of different spin states of such a multiconfigurational system as tetraradical(oid).

For an appropriate description of an electronic structure of tetraradical(oid), a qualitatively correct wave function can be obtained by multiconfigurational SCF (MCSCF) methods such as Complete Active Space SCF (CASSCF), also known as Full Optimized Reaction Space (FORS),[8–15] which describes the so-called static/non-dynamic correlation. HF, CASSCF and CASCI (explained below) calculations were performed using The General Atomic and Molecular Electronic Structure System (GAMESS) version 2016 (R1).[16] For UHF and UKS DFT benchmark given in Section S4 as well as for CASSCF and CASCI calculations, Dunning's correlation-consistent double zeta basis set cc-pVDZ was used.[17] CAS Configuration Interaction (CASCI) is the single-variable mode of CASSCF, in which molecular orbitals coefficients are not being optimized but only CI coefficients. DFT and UHF benchmark given in Section S4 was done with GAMESS version 2023 (R2)[18] due to availability of building custom exchange-correlation functionals. Improved descriptions including dynamic electron correlation require expensive multi-reference perturbation (MRPT) or multi-reference configuration-interaction (MRCI) calculations, which considering the size of the system – CASSCF and CASCI with (16,16) active space – cannot be computed. Even for (4,4) minimal active space (considering that, by definition, only 4 unpaired electrons can be described) 681 external molecular orbitals that remain, considering the basis set and active space, are too many to include. Only including up to 30 or 40 external orbitals captures only negligible part of the correlation energy. This was checked by the corresponding Multireference Moller-Plesset second order Perturbation Theory (MRMP2)[19–24] calculations, which showed by more than 100 factors of correlation energy correction than what MRCI showed with only including 30-40 external orbitals. Therefore, the only computationally practical method for studying the system of the size that is presented in this work is MRMP2. Nonetheless, since the results from MRMP2 gave same qualitative picture as CASCI and CASSCF calculations, they are not included nor discussed in this work due to redundancy.

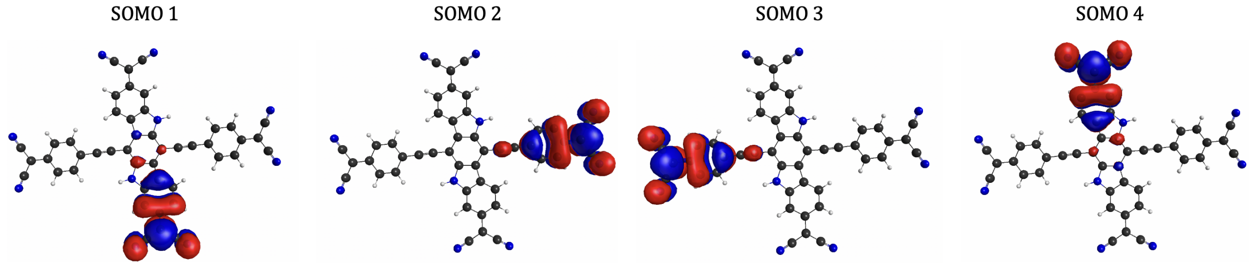

Figure S2. Localized singly occupied molecular orbitals of converged solution of ROHF quintet obtained from starting with converged UHF quintet NOs guess.

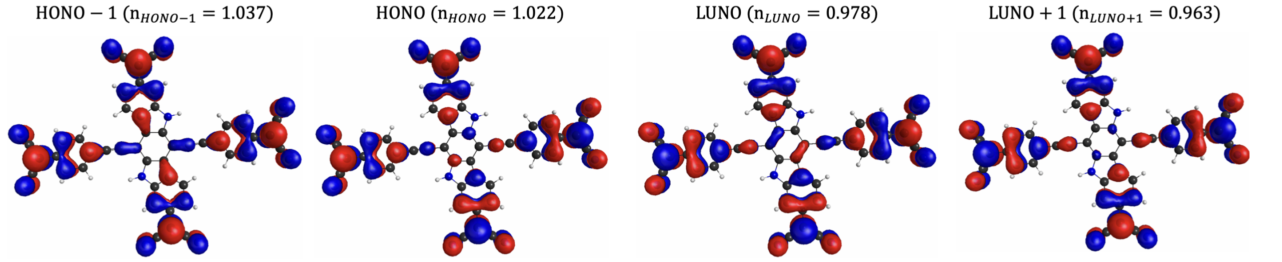

Figure S3. UHF SONOs of converged solution of UHF singlet obtained from starting with converged ROHF quintet localized frontier orbitals guess.

S2.3. Check of the qualitative electronic structure of the ground state with HF

At first, RHF and UHF calculations were done for a singlet state to determine whether or not the ground state electronic structure of this potential tetraradical(oid) is open-shell (justified by the resonance structures of the compound given in Figure S1). It turned out that the UHF broken-symmetry singlet has much lower energy (by 189.6 kcal/mol) than the RHF solution. Also, occupation numbers of Natural Orbitals (NOs) indicate that there are four Singly Occupied NOs (SONOs), which implies four unpaired electrons, meaning that molecule is a tetraradical(oid). NOs are eigenvectors of the first-order density matrix operator, and a corresponding eigenvalue to the particular NO is its occupation number[25] (denoted as n_{NO}). The Highest Occupied NO (HONO) is defined as the orbital which has the lowest occupation number n_{NO} among NOs with $n_{NO} \geq 1$. Lowest Unoccupied NO (LUNO) is defined as the orbital which has the highest n_{NO} among NOs with $n_{NO} \leq 1$. In UHF, $n_{HONO-i} + n_{LUNO+i} = 2.000$, which is also manifested usually in CASSCF and CASCI natural orbitals. Since it is important to study the molecule as having potentially 4 persistent unpaired electrons, broken-symmetry solution was not simply obtained by mixing Highest Occupied Molecular Orbital (HOMO) and Lowest Unoccupied Molecular Orbital (LUMO), but with the following procedure. Firstly, UHF quintet calculation was run, then UHF NOs were taken as guess for Restricted Open-Shell Hartree-Fock (ROHF) quintet calculation. Then Pipek-Mezey localization[26] was done in order to localize converged ROHF open-shell/singly occupied orbitals. Having checked the localization, it showed that each Singly Occupied Molecular Orbital (SOMO) was localized on each particular spin center (see Figure S2). After localization, the orbitals were ordered in order to obtain a singlet of the type $|\alpha(1)\alpha(2)\beta(3)\beta(4)\rangle$, where orbitals are assigned as follows from the Figure S2: SOMO 1 $\equiv$ 1, SOMO 2 $\equiv$ 2, SOMO 3 $\equiv$ 3, SOMO 4 $\equiv$ 4. Thus, both *subsystems* $\boldsymbol{S}$ and $\boldsymbol{L}$ are initiated as having opposite spins for their respective two unpaired electrons. Resulting UHF singlet NOs with their occupation numbers are given in Figure S3. This showed that the molecular electronic structure of the $\boldsymbol{LS}$ is tetraradical(oid) with spin-opposite unpaired electrons for each substructure $\boldsymbol{S}$ and $\boldsymbol{L}$. NOs occupation number complementarity and still not exactly being 1.000 (but very close to it) shows that the $\boldsymbol{LS}$ is indeed a tetraradical(oid) with very high tetraradical character. Although HF is sufficient to qualitatively gauge the ground state character of the electronic structure of $\boldsymbol{LS}$, UHF states are not pure. To describe the electronic structure and distribution of states of the possible tetraradical(oid) qualitatively correctly, we need to employ multiconfigurational calculations such as CASSCF and CASCI.

S2.4. CASSCF and CASCI calculation details

Having determined that $\boldsymbol{LS}$ is a potential tetraradical(oid), UHF quintet calculation was run to obtain UHF NOs, which includes 4 SONOs. Therefore, to study a potential tetraradical(oid), one of the best guess orbitals are UHF quintet NOs. The NOs were selected in the active space based on their NO occupation numbers with reasonable cutoff so as to include all the degenerate relevant orbitals and maintain the electronic problem tractable.[27] Hence, based on these limits, the largest active space that was used for MCSCF calculations was 16 orbitals

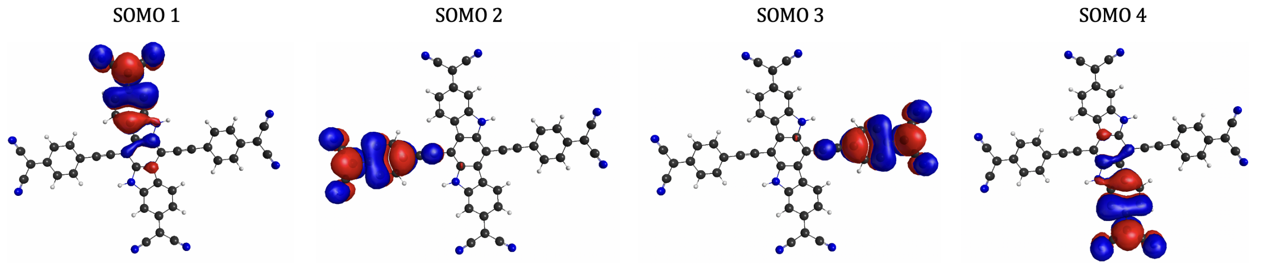

Figure S4. Localized singly occupied molecular orbitals of converged solution of CASSCF(16,16) quintet obtained from starting with converged UHF quintet NOs guess.

with 16 electrons – (16,16). Note that multiconfigurational wavefunction can be obtained for symmetry-adapted (if guess orbitals are not localized) or C_1 point group states (if guess orbitals are localized). With $S_z = 0$ determinant basis, (4,4) and (16,16) CASSCF calculations were run for 8 states from the UHF quintet NOs guess centered around HONO and LUNO, thus (4,4) representing minimal model space for 4 unpaired electrons (thus tetraradical(oid)). Also, to demonstrate the persistence of tetraradical spin states, the CASSCF(10,10) and CASSCF(14,14) calculations were run with UHF triplet NOs guess. We found with every size of active space that there are six states that are very close to each other in the ranges from which 603 cm^{-1} is a maximum.

After determining 6 spin states of tetraradical(oid) $\boldsymbol{LS}$, and seeing that energies of these two singlets, three triplets and one quintet are very close, converged CASSCF(16,16) quintet optimized orbitals were taken, and (4,4) model frontier subspace (those that represent 4 SOMOs) was localized within (16,16) active space and used as a guess for CASCI(4,4) and CASCI(16,16) calculations. Having inspected that localization resulted each SOMOs being assigned to particular center as shown in Figure S4, we can also analyze the determinantal build of the corresponding wavefunctions of the spin states determined by CASCI. More than six states were determined with $S_z = 0$ determinant basis. For (4,4) model space, these determinants are $|\alpha(1)\alpha(2)\beta(3)\beta(4)\rangle$, $|\beta(1)\beta(2)\alpha(3)\alpha(4)\rangle$, $|\alpha(1)\beta(2)\alpha(3)\beta(4)\rangle$, $|\beta(1)\alpha(2)\beta(3)\alpha(4)\rangle$, $|\alpha(1)\beta(2)\beta(3)\alpha(4)\rangle$, $|\beta(1)\alpha(2)\alpha(3)\beta(4)\rangle$. It turned out from CASCI that the ordering of states is the following: $S_0, T_0, T_1, S_1, T_2, Q_0$ within range of 460.9 cm^{-1}. Orbital numbering is as follows from Figure S4: SOMO 1 $\equiv$ 1, SOMO 2 $\equiv$ 2, SOMO 3 $\equiv$ 3, SOMO 4 $\equiv$ 4. Thus, CI coefficients for each determinantal contribution can be translated into electron density distribution of spin centers. The existence of low-energy quasi-degenerate (magnetic scale difference) states with all the possible spin multiplicities for a tetraradical electronic structure shows that our molecule has 4 unpaired electrons. Thus, $\boldsymbol{LS}$ is a tetraradical(oid).

S2.5. Broken-symmetry calculations with HF and DFT

For broken-symmetry calculations with HF and DFT, converged CASSCF(16,16) quintet orbitals were taken and (4,4) frontier subspace localized as shown in Figure S4. These orbitals are assigned as for previous CASCI calculations in Section S2.4. Then α and β sets of localized guess orbitals were reordered to model the determinants for each spin states such that $S = S_z$. Since, in principle, $|\alpha\alpha\alpha\alpha\rangle$ is equivalent to $|\beta\beta\beta\beta\rangle$ because both describe all-spin-parallel determinants, this equivalence and molecular symmetry have been used to define unique determinants for each $S = S_z$ subspace. Unique singlet determinants are $|\alpha(1)\alpha(2)\beta(3)\beta(4)\rangle$, $|\alpha(1)\beta(2)\beta(3)\alpha(4)\rangle$, $|\alpha(1)\beta(2)\alpha(3)\beta(4)\rangle$, unique triplet determinants are $|\alpha(1)\alpha(2)\alpha(3)\beta(4)\rangle$, $|\alpha(1)\alpha(2)\beta(3)\alpha(4)\rangle$, and unique quintet determinant is $|\alpha(1)\alpha(2)\alpha(3)\alpha(4)\rangle$.

S3. Calculated results

S3.1. CASSCF and CASCI results

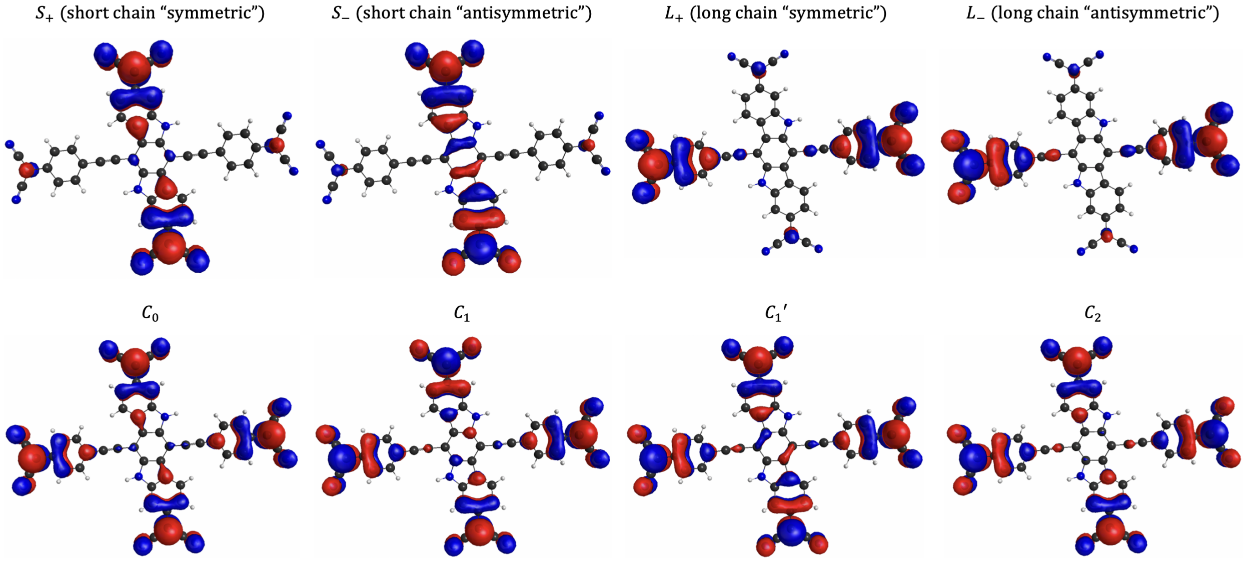

Figure S5. Symbolic assignations to singly cccupied natural orbitals that appear for frontier orbitals in the CASSCF and CASCI solutions.

For all the calculation results, the 8 types of orbitals are obtained for frontier singly occupied molecular (or natural) orbitals. If we assign symbols, we can categorize each spin state with these orbitals, their symmetry and

Table S1. CASSCF(4,4) states determined by state-specific calculations. For all the calculations, UHF quintet NOs were used as initial guess. Under HONO, LUNO, etc. three columns represent symmetry of the particular orbital, its symbolic representation according to Figure S5 and NO occupation number.

State	Symmetry	Energy (a.u.)	CASSCF NOs symmetry, identity and occupation number.												ΔE from G. S. (cm^{-1})
			HONO − 1			HONO			LUNO			LUNO + 1			
S_0	A_g	-2295.6577083553	A_u	S_+	1.155	A_u	L_+	1.038	B_g	L_-	0.961	B_g	S_-	0.846	0.00
T_0	B_u	-2295.6575974661	A_u	S_+	1.155	B_g	L_-	1.001	A_u	L_+	0.998	B_g	S_-	0.846	24.34
S_1	A_g	-2295.6565732806	A_u	C_0	1.046	B_g	C_1	1.011	B_g	$C_1^{'}$	0.982	A_u	C_2	0.961	249.12
T_1	B_u	-2295.6565606467	A_u	L_+	1.046	B_g	S_-	0.999	A_u	S_+	0.998	B_g	L_-	0.957	251.89
T_2	A_g	-2295.6565364226	A_u	C_0	1.033	B_g	C_1	1.019	B_g	$C_1^{'}$	0.981	A_u	C_2	0.967	257.21
Q_0	A_g	-2295.6564170504	B_g	L_-	1.000	A_u	C_0	1.000	A_u	C_2	1.000	B_g	S_-	1.000	283.41

Table S2. CASSCF(10,10) states determined by state-specific calculations. For all the calculations, UHF triplet NOs were used as initial guess. Under HONO, LUNO, etc. three columns represent symmetry of the particular orbital, its symbolic representation according to Figure S5 and NO occupation number.

State	Symmetry	Energy (a.u.)	CASSCF NOs symmetry, identity and occupation number												ΔE from G. S. (cm^{-1})
			HONO − 1			HONO			LUNO			LUNO + 1			
S_0	A_g	-2295.7060045679	A_u	S_+	1.146	A_u	L_+	1.046	B_g	L_-	0.951	B_g	S_-	0.857	0.00
T_0	B_u	-2295.7054825012	A_u	S_+	1.135	A_u	L_+	0.999	B_g	L_-	0.998	B_g	S_-	0.867	114.58
T_1	B_u	-2295.7047258955	A_u	L_+	1.056	A_u	S_+	1.018	B_g	S_-	0.979	B_g	L_-	0.947	280.64
T_2	A_g	-2295.7044921946	A_u	C_0	1.040	B_g	C_1	1.021	B_g	$C_1^{'}$	0.977	A_u	C_2	0.961	331.93
S_1	A_g	-2295.7044427823	A_u	C_0	1.052	B_g	C_1	0.998	B_g	$C_1^{'}$	0.984	A_u	C_2	0.966	342.77
Q_0	A_g	-2295.7043163461	A_u	C_0	1.002	B_g	S_-	1.000	A_u	C_2	1.000	B_g	L_-	0.998	370.52

Table S3. CASSCF(14,14) states determined by state-specific calculations. For all the calculations, UHF triplet NOs were used as initial guess. Under HONO, LUNO, etc. three columns represent symmetry of the particular orbital, its symbolic representation according to Figure S5 and NO occupation number.

State	Symmetry	Energy (a.u.)	CASSCF NOs symmetry, identity and occupation number												ΔE from G. S. (cm^{-1})
			HONO − 1			HONO			LUNO			LUNO + 1			
S_0	A_g	-2295.7404329632	A_u	S_+	1.192	A_u	L_+	1.051	B_g	L_-	0.947	B_g	S_-	0.813	0.00
T_0	B_u	-2295.7398508757	A_u	S_+	1.190	B_g	L_-	0.999	A_u	L_+	0.998	B_g	S_-	0.816	127.75
T_1	B_u	-2295.7384006857	A_u	L_+	1.064	A_u	S_+	1.013	B_g	S_-	0.986	B_g	L_-	0.940	446.03
T_2	A_g	-2295.7381616533	A_u	C_0	1.046	B_g	C_1	1.026	B_g	$C_1^{'}$	0.974	A_u	C_2	0.957	498.49
S_1	A_g	-2295.7380978691	A_u	C_0	1.059	B_g	C_1	1.001	B_g	$C_1^{'}$	0.981	A_u	C_2	0.961	512.49
Q_0	A_g	-2295.7379321028	A_u	C_0	1.003	B_g	S_-	1.001	A_u	C_2	1.000	B_g	L_-	0.998	548.88

Table S4. CASSCF(16,16) states determined by state-specific calculations. For all the calculations, UHF quintet NOs were used as initial guess. Under HONO, LUNO, etc. three columns represent symmetry of the particular orbital, its symbolic representation according to Figure S5 and NO occupation number.

State	Symmetry	Energy (a.u.)	CASSCF NOs symmetry, identity and occupation number												ΔE from G. S. (cm^{-1})
			HONO − 1			HONO			LUNO			LUNO + 1			
S_0	A_g	-2295.7831909607	A_u	S_+	1.221	A_u	L_+	1.043	B_g	L_-	0.955	B_g	S_-	0.781	0.00
T_0	B_u	-2295.7829323238	A_u	S_+	1.222	B_g	L_-	1.000	A_u	L_+	0.998	B_g	S_-	0.780	56.76
T_1	B_u	-2295.7807627335	A_u	L_+	1.058	A_u	S_+	0.997	B_g	S_-	0.995	B_g	L_-	0.948	532.93
S_1	A_g	-2295.7807543493	A_u	C_0	1.059	B_g	C_1	1.011	B_g	$C_1^{'}$	0.977	A_u	C_2	0.951	534.77
T_2	A_g	-2295.7806889597	A_u	C_0	1.043	B_g	C_1	1.021	B_g	$C_1^{'}$	0.975	A_u	C_2	0.959	549.13
Q_0	A_g	-2295.7804446812	A_u	C_0	1.002	A_u	C_2	0.999	B_g	L_-	0.999	B_g	S_-	0.997	602.74

Table S5. CASCI(4,4) from CASSCF(16,16) quintet converged orbitals as initial guess. Under HONO, LUNO, etc. three columns represent symmetry of the particular orbital, its symbolic representation according to Figure S5 and NO occupation number.

State	Symmetry	Energy (a.u.)	CASCI NOs occupancy, identity and symmetry												ΔE from G. S. (cm^{-1})
			HONO − 1			HONO			LUNO			LUNO + 1			
S_0	A	-2295.6320879374	A	S_+	1.179	A	L_+	1.050	A	L_-	0.947	A	S_-	0.825	0.00
T_0	A	-2295.6318220969	A	S_+	1.175	A	L_-	1.001	A	L_+	0.996	A	S_-	0.828	58.35
T_1	A	-2295.6303232335	A	L_+	1.073	A	S_-	0.998	A	S_+	0.995	A	L_-	0.935	387.31
S_1	A	-2295.6303058597	A	C_0	1.075	A	C_1	1.018	A	$C_1^{'}$	0.974	A	C_2	0.933	391.12
T_2	A	-2295.6302429049	A	C_0	1.054	A	C_1	1.027	A	$C_1^{'}$	0.973	A	C_2	0.946	404.94
Q_0	A	-2295.6299880625	A	D_0 [a]	1.000	A	D_1	1.000	A	D_2	1.000	A	D_3	1.000	460.87

[a] D_0, D_1, D_2, D_3 means **distorted** orbitals. Overall electron density corresponds to the same as if they were from C_0, C_1, $C_1^{'}$, C_2 orbitals.

Table S6. CASCI(16,16) from CASSCF(16,16) quintet converged orbitals as initial guess. Under HONO, LUNO, etc. three columns represent symmetry of the particular orbital, its symbolic representation according to Figure S5 and NO occupation number.

State	Symmetry	Energy (a.u.)	CASCI NOs occupancy, identity and symmetry.												ΔE from G. S. (cm^{-1})
			HONO − 1			HONO			LUNO			LUNO + 1			
S_0	A	-2295.7822275550	A	S_+	1.136	A	L_+	1.034	A	L_-	0.964	A	S_-	0.865	0.00
T_0	A	-2295.7819983012	A	S_+	1.134	A	L_-	1.000	A	L_+	0.998	A	S_-	0.867	50.32
T_1	A	-2295.7807288268	A	L_+	1.050	A	S_+	0.996	A	S_-	0.996	A	L_-	0.956	328.93
S_1	A	-2295.7807093620	A	C_0	1.054	A	C_1	1.013	A	C_1'	0.977	A	C_2	0.955	333.20
T_2	A	-2295.7806591096	A	C_0	1.040	A	C_1	1.019	A	C_1'	0.978	A	C_2	0.962	344.23
Q_0	A	-2295.7804446450	A	C_0	1.002	A	C_2	0.999	A	L_-	0.999	A	S_-	0.997	391.30

Table S7. CASCI(4,4) determinant contributions for 6 near-degenerate tetraradical(oid) spin states. These states are obtained for $S_z = 0$ basis from CASSCF(16,16) converged quintet localized (in (4,4) SOMOs subspace) initial guess orbitals.

	S_0	T_0	T_1	S_1	T_2	Q_0
ΔE from ground state (cm^{-1})	0.00	58.35	387.31	391.12	404.94	460.87
$\vert\alpha\alpha\beta\beta\rangle$	0.4342037680	0.4288307452	-0.5602426962	-0.3775905620	0.00000000	0.4082482905
$\vert\beta\beta\alpha\alpha\rangle$	0.4342037680	-0.4288307452	0.5602426962	-0.3775905620	0.00000000	0.4082482905
$\vert\alpha\beta\alpha\beta\rangle$	0.5429373792	-0.5584143400	-0.4303043443	0.1886158786	0.00000000	-0.4082482905
$\vert\beta\alpha\beta\alpha\rangle$	0.5429373792	0.5584143400	0.4303043443	0.1886158786	0.00000000	-0.4082482905
$\vert\alpha\beta\beta\alpha\rangle$	0.1087336111	0.00000000	0.00000000	0.5662064406	-0.7064532311	0.4082482905
$\vert\beta\alpha\alpha\beta\rangle$	0.1087336111	0.00000000	0.00000000	0.5662064406	0.7064532311	0.4082482905
Sum of coefficients $\sum_{I\in CAS} c_I^2$	0.9903	0.9914	0.9981	0.9975	0.9982	1.0000

Table S8. CASCI(16,16) determinant contributions for 6 near-degenerate tetraradical(oid) spin states. These states are obtained for $S_z = 0$ basis from CASSCF(16,16) converged quintet localized (in (4,4) SOMOs subspace) initial guess orbitals. Explicit determinant coefficients are given as fraction of 1, while weight summations are given as a %. "Anti-Heis." means determinants that are not neutral in minimal (4,4) active space.

	S_0	T_0	T_1	S_1	T_2	Q_0
ΔE from ground state (cm^{-1})	0.00	50.32	328.93	333.20	344.23	391.30
$\vert 222222\alpha\alpha\beta\beta000000\rangle$	0.3686326655	0.3636133199	0.4770549800	-0.3221829822	0.00000000	0.3465629793
$\vert 222222\beta\beta\alpha\alpha000000\rangle$	0.3686326655	-0.3636133191	-0.4770549462	-0.3221830374	0.00000000	0.3465629754
$\vert 222222\alpha\beta\alpha\beta000000\rangle$	0.4630752531	-0.4765339505	0.3641058319	0.1585111406	0.00000000	-0.3465629484
$\vert 222222\beta\alpha\beta\alpha000000\rangle$	0.4630752524	0.4765339507	-0.3641058476	0.1585110993	.00000000	-0.3465629517
$\vert 222222\alpha\beta\beta\alpha000000\rangle$	0.0944425744	0.00000000	0.00000000	0.4806942354	0.6000760328	0.3465628835
$\vert 222222\beta\alpha\alpha\beta000000\rangle$	0.0944425739	0.00000000	0.00000000	0.4806942396	-0.6000760319	0.3465628794
Heis. term weights $\sum_{I\in CAS(4,4)} c_I^2$	71.8496	71.8599	72.0309	71.9989	72.0182	72.0635
Anti-Heis. weights $\sum_{I\in N.H.} c_I^2$	4.7131	4.7834	4.7099	4.5592	5.0854	4.1317
S-S* $\sum_{I\in S-S^*} c_I^2$	3.3035	3.3002	3.2060	3.2404	3.2306	3.1933
L-L* $\sum_{I\in L-L^*} c_I^2$	6.3218	6.3475	6.3740	6.4315	6.4194	6.3519
L-S* $\sum_{I\in L-S^*} c_I^2$	0.0281	0.0294	0.0298	0.0000	0.0000	0.0631
S-L* $\sum_{I\in S-L^*} c_I^2$	0.0514	0.0540	0.0720	0.0000	0.0298	0.0599
S,S-S*,S* $\sum_{I\in S,S-S^*,S^*} c_I^2$	1.9513	1.9514	1.9535	1.9618	1.9642	1.9556
S,S-L*,S* $\sum_{I\in S,S-L^*,S^*} c_I^2$	0.0000	0.0000	0.0000	0.0000	0.0052	0.0000
S,S-L*,L* $\sum_{I\in S,S-L^*,L^*} c_I^2$	0.0000	0.0000	0.0000	0.0000	0.0000	0.0000
L,L-L*,L* $\sum_{I\in L,L-L^*,L^*} c_I^2$	6.4523	6.6063	6.6231	6.4147	6.7900	6.4322
L,L-L*,S* $\sum_{I\in L,L-L^*,S^*} c_I^2$	0.0000	0.0000	0.0000	0.0000	0.0000	0.0000
L,L-S*,S* $\sum_{I\in L,L-S^*,S^*} c_I^2$	0.0000	0.0000	0.0000	0.0000	0.0000	0.0000
L,S-S*,S* $\sum_{I\in L,S-S^*,S^*} c_I^2$	0.0000	0.0000	0.0000	0.0000	0.0000	0.0000
L,S-L*,S* $\sum_{I\in L,S-L^*,S^*} c_I^2$	0.0734	0.0734	0.0706	0.0000	0.0000	0.0400
L,S-L*,L* $\sum_{I\in L,S-L^*,L^*} c_I^2$	0.0000	0.0000	0.0000	0.0000	0.0000	0.0000
Triple excitations	0.0539	0.0546	0.0535	0.0000	0.0000	0.0000
Other contributions	0.0078	0.0135	0.0135	0.0137	0.0269	0.0000
Total $\sum_{I\in CAS(16,16)} c_I^2$	94.8062	95.0737	95.1368	94.6203	95.5697	94.2913
Number of Dets.	1074	990	1004	1088	690	1112

their NO occupation numbers. These assignations are given in Figure S5. CASSCF(4,4) calculations results are given in Table S1, CASSCF(10,10) calculations results are given in Table S2, CASSCF(14,14) calculations results in Table S3, and CASSCF(16,16) calculations results in Table S4. As we see from these results, we have 6 states

which are very close to each other in energy. Also, the properties of corresponding spin states are very similar in calculations across different sizes of active space. The maximum range is, as mentioned before, 603 cm^{-1}. This means that ***LS*** is a magnetic molecule as the transition energies between its different spin states are very small.

To better understand the properties of the molecule, we ran CASCI(4,4) and CASCI(16,16) from CASSCF(16,16) quintet converged orbitals with 4 SOMOs localized. CI coefficients of each determinantal contribution can be translated into electron density distribution of spin centers, because of localized guess. Results for CASCI(4,4) are given in Table S5 and results for CASCI(16,16) are given in Table S6. To gain further insight into the electronic structure of these spin states of the tetraradical(oid), we can analyze determinantal composition of wavefunction for each spin state. This kind of analysis for CASCI(4,4) calculation is given in Table S7 and for CASCI(16,16) calculation is given in Table S8.

S3.2. Results from Heisenberg-Dirac-van Vleck model and effective Hamiltonian theory

Heisenberg model describes open-shell systems as particle-per-site model of spin centers.[28] The general structure of this model is well known in the chemistry and (especially) molecular or solid-state physics community. The model spin Hamiltonian is called Heisenberg-Dirac-van Vleck (HDVV) Hamiltonian[28–30] and corresponding energy expressions of its eigenvalues are expressed in terms of two-body magnetic coupling constants $J_{12}, J_{13}, J_{14}, J_{23}, J_{24}, J_{34}$. We can make a matrix representation of the HDVV hamiltonian as follows from Eq. S1:[31]

$$\hat{H}_{HDVV} = -\sum_{i<j} J_{ij}\hat{\boldsymbol{S}}_i \cdot \hat{\boldsymbol{S}}_j \tag{S1}$$

For a 4-site model, this Hamiltonian can be expanded as follows from Eq. S2:

$$\hat{H}_{HDVV} = -J_{12}\hat{\boldsymbol{S}}_1 \cdot \hat{\boldsymbol{S}}_2 - J_{13}\hat{\boldsymbol{S}}_1 \cdot \hat{\boldsymbol{S}}_3 - J_{14}\hat{\boldsymbol{S}}_1 \cdot \hat{\boldsymbol{S}}_4 - J_{23}\hat{\boldsymbol{S}}_2 \cdot \hat{\boldsymbol{S}}_3 - J_{24}\hat{\boldsymbol{S}}_2 \cdot \hat{\boldsymbol{S}}_4 - J_{34}\hat{\boldsymbol{S}}_3 \cdot \hat{\boldsymbol{S}}_4 \tag{S2}$$

Where J_{ij} is the exchange-coupling constant between $\hat{\boldsymbol{S}}_i$ and $\hat{\boldsymbol{S}}_j$ localized spin moments. According to the adopted definition of this model Hamiltonian in Eq. S1, the positive value of the exchange-coupling constant corresponds to an antiferromagnetic interaction between $\hat{\boldsymbol{S}}_i$ and $\hat{\boldsymbol{S}}_j$ magnetic moments, while negative value corresponds to the ferromagnetic interaction between these magnetic moments i and j (anti-parallel and parallel spin alignments, respectively).

In order to build up this Hamiltonian matrix in the basis that is appropriate, we can choose $S_z = 0$ subspace for which all spin states that a tetraradical(oid) could have (singlet, triplet and quintet) can be represented with expansion of 6 determinants, which are all neutral determinants in (4,4) active space with $S_z = 0$. Matrix elements can be found by applying the following relation expressed in Eq. S3[31] as follows:

$$\hat{\boldsymbol{S}}_i \cdot \hat{\boldsymbol{S}}_j = \frac{1}{2}\hat{\boldsymbol{S}}_{i+}\hat{\boldsymbol{S}}_{j-} + \frac{1}{2}\hat{\boldsymbol{S}}_{i-}\hat{\boldsymbol{S}}_{j+} + \hat{\boldsymbol{S}}_{iz}\hat{\boldsymbol{S}}_{jz} \tag{S3}$$

Where $\hat{\boldsymbol{S}}_{i+}$ and $\hat{\boldsymbol{S}}_{j-}$ are ladder operators applied to particular spin center i and j, respectively. Therefore, by application of these operators, we can find matrix elements of the HDVV Hamiltonian in the basis of the $S_z = 0$ determinants. The basis is ordered as follows: $|\alpha\alpha\beta\beta\rangle$, $|\beta\beta\alpha\alpha\rangle$, $|\alpha\beta\alpha\beta\rangle$, $|\beta\alpha\beta\alpha\rangle$, $|\alpha\beta\beta\alpha\rangle$, $|\beta\alpha\alpha\beta\rangle$. For example, to find the part of element $\hat{H}_{HDVV}$(3,3) for J_{12} term, we can apply this expansion as shown in Eq. S4:

$$\begin{aligned}
\hat{H}_{HDVV}(3,3 \text{ for } J_{12}) &= -J_{12}\left\langle \alpha\beta\alpha\beta \,\middle|\, \hat{\boldsymbol{S}}_1\hat{\boldsymbol{S}}_2 \,\middle|\, \alpha\beta\alpha\beta \right\rangle \\
&= -J_{12}\left\langle \alpha\beta\alpha\beta \,\middle|\, \frac{1}{2}\hat{\boldsymbol{S}}_{1+}\hat{\boldsymbol{S}}_{2-} + \frac{1}{2}\hat{\boldsymbol{S}}_{1-}\hat{\boldsymbol{S}}_{2+} + \hat{\boldsymbol{S}}_{1z}\hat{\boldsymbol{S}}_{2z} \,\middle|\, \alpha\beta\alpha\beta \right\rangle \\
&= -J_{12}\left\langle \alpha\beta\alpha\beta \,\middle|\, \frac{1}{2}\cdot(-\frac{1}{2}) \,\middle|\, \alpha\beta\alpha\beta \right\rangle \\
&= \frac{J_{12}}{4}\left\langle \alpha\beta\alpha\beta \,|\, \alpha\beta\alpha\beta \right\rangle = \frac{J_{12}}{4}
\end{aligned} \tag{S4}$$

Because 6 determinants of the $S_z = 0$ form an orthonormal set. By application of these operators, we obtain the following HDVV Hamiltonian matrix as given in Eq. S5.

$$\hat{H}_{HDVV} = \begin{pmatrix}
-\frac{J_{12}}{4}+\frac{J_{13}}{4}+\frac{J_{14}}{4}+\frac{J_{23}}{4}+\frac{J_{24}}{4}-\frac{J_{34}}{4} & 0 & -\frac{J_{23}}{2} & -\frac{J_{14}}{2} & -\frac{J_{24}}{2} & -\frac{J_{13}}{2} \\
0 & -\frac{J_{12}}{4}+\frac{J_{13}}{4}+\frac{J_{14}}{4}+\frac{J_{23}}{4}+\frac{J_{24}}{4}-\frac{J_{34}}{4} & -\frac{J_{14}}{2} & -\frac{J_{23}}{2} & -\frac{J_{13}}{2} & -\frac{J_{24}}{2} \\
-\frac{J_{23}}{2} & -\frac{J_{14}}{2} & \frac{J_{12}}{4}-\frac{J_{13}}{4}+\frac{J_{14}}{4}+\frac{J_{23}}{4}-\frac{J_{24}}{4}+\frac{J_{34}}{4} & 0 & -\frac{J_{34}}{2} & -\frac{J_{12}}{2} \\
-\frac{J_{14}}{2} & -\frac{J_{23}}{2} & 0 & \frac{J_{12}}{4}-\frac{J_{13}}{4}+\frac{J_{14}}{4}+\frac{J_{23}}{4}-\frac{J_{24}}{4}+\frac{J_{34}}{4} & -\frac{J_{12}}{2} & -\frac{J_{34}}{2} \\
-\frac{J_{24}}{2} & -\frac{J_{13}}{2} & -\frac{J_{34}}{2} & -\frac{J_{12}}{2} & \frac{J_{12}}{4}+\frac{J_{13}}{4}-\frac{J_{14}}{4}-\frac{J_{23}}{4}+\frac{J_{24}}{4}+\frac{J_{34}}{4} & 0 \\
-\frac{J_{13}}{2} & -\frac{J_{24}}{2} & -\frac{J_{12}}{2} & -\frac{J_{34}}{2} & 0 & \frac{J_{12}}{4}+\frac{J_{13}}{4}-\frac{J_{14}}{4}-\frac{J_{23}}{4}+\frac{J_{24}}{4}+\frac{J_{34}}{4}
\end{pmatrix} \tag{S5}$$

If we note that the molecule has C_{2h} point group symmetry and refer to the notation of SOMOs in Figure S4, we can note that interactions between magnetic centers 1 and 2 is equivalent to those between 3 and 4, and interactions between magnetic centers 1 and 3 is equivalent to the interaction between centers 2 and 4 (due to C_2 rotational symmetry). Therefore, we create the following notation for further simplification: $J_{12} = J_{34} = J_a$, $J_{13} = J_{24} = J_b$. In addition, considering the previous notation of $\boldsymbol{S}$ and $\boldsymbol{L}$, we create the following notations: $J_{14} = J_S$ and $J_{23} = J_L$. Hence, the HDVV Hamiltonian is simplified as given in Eq. S6:

$$\hat{H}_{HDVV} = \begin{pmatrix} -\frac{J_a}{2}+\frac{J_S}{4}+\frac{J_L}{4}+\frac{J_b}{2} & 0 & -\frac{J_L}{2} & -\frac{J_S}{2} & -\frac{J_b}{2} & -\frac{J_b}{2} \\ 0 & -\frac{J_a}{2}+\frac{J_S}{4}+\frac{J_L}{4}+\frac{J_b}{2} & -\frac{J_S}{2} & -\frac{J_L}{2} & -\frac{J_b}{2} & -\frac{J_b}{2} \\ -\frac{J_L}{2} & -\frac{J_S}{2} & \frac{J_a}{2}+\frac{J_S}{4}+\frac{J_L}{4}-\frac{J_b}{2} & 0 & -\frac{J_a}{2} & -\frac{J_a}{2} \\ -\frac{J_S}{2} & -\frac{J_L}{2} & 0 & \frac{J_a}{2}+\frac{J_S}{4}+\frac{J_L}{4}-\frac{J_b}{2} & -\frac{J_a}{2} & -\frac{J_a}{2} \\ -\frac{J_b}{2} & -\frac{J_b}{2} & -\frac{J_a}{2} & -\frac{J_a}{2} & \frac{J_a}{2}-\frac{J_S}{4}-\frac{J_L}{4}+\frac{J_b}{2} & 0 \\ -\frac{J_b}{2} & -\frac{J_b}{2} & -\frac{J_a}{2} & -\frac{J_a}{2} & 0 & \frac{J_a}{2}-\frac{J_S}{4}-\frac{J_L}{4}+\frac{J_b}{2} \end{pmatrix} \tag{S6}$$

We can diagonalize this Hamiltonian to find its eigenvalues and eigenvectors. This results in the following eigenvalues as given in Eq. S7 and Eq. S8, with unnormalized eigenvectors in Eq. S9.

$$\begin{aligned} E_0 &= -\frac{J_S + J_L + 2J_b + 2J_a}{4} & E_1 &= -\frac{2X - J_S - J_L}{4} \\ E_2 &= \frac{2X + J_S + J_L}{4} & E_3 &= -\frac{2Y - J_S - J_L - 2J_b - 2J_a}{4} \\ E_4 &= \frac{2Y + J_S + J_L + 2J_b + 2J_a}{4} & E_5 &= -\frac{J_S + J_L - 2J_b - 2J_a}{4} \end{aligned} \tag{S7}$$

Where

$$\begin{aligned} X &\equiv \sqrt{J_S^2 + J_L^2 + J_a^2 + J_b^2 - 2J_SJ_L - 2J_aJ_b} \\ Y &\equiv \sqrt{J_S^2 + J_L^2 + 4J_a^2 + 4J_b^2 + (2J_L - 2J_b - 2J_a)J_S - (2J_a + 2J_b)J_L - 4J_aJ_b} \end{aligned} \tag{S8}$$

$$\begin{aligned} \psi_0 &= |\alpha\alpha\beta\beta\rangle + |\beta\beta\alpha\alpha\rangle + |\alpha\beta\alpha\beta\rangle + |\beta\alpha\beta\alpha\rangle + |\alpha\beta\beta\alpha\rangle + |\beta\alpha\alpha\beta\rangle \\ \psi_1 &= |\alpha\alpha\beta\beta\rangle - |\beta\beta\alpha\alpha\rangle - \frac{X - J_a + J_b}{J_S - J_L}|\alpha\beta\alpha\beta\rangle + \frac{X - J_a + J_b}{J_S - J_L}|\beta\alpha\beta\alpha\rangle \\ \psi_2 &= |\alpha\alpha\beta\beta\rangle - |\beta\beta\alpha\alpha\rangle - \frac{X + J_a - J_b}{J_S - J_L}|\alpha\beta\alpha\beta\rangle + \frac{X + J_a - J_b}{J_S - J_L}|\beta\alpha\beta\alpha\rangle \\ \psi_3 &= |\alpha\alpha\beta\beta\rangle + |\beta\beta\alpha\alpha\rangle + \frac{Y - 2J_a + 2J_b}{J_S + J_L - 2J_b}|\alpha\beta\alpha\beta\rangle + \frac{Y - 2J_a + 2J_b}{J_S + J_L - 2J_b}|\beta\alpha\beta\alpha\rangle - \\ &\quad - \frac{Y + J_S + J_L - 2J_a}{J_S + J_L - 2J_b}|\alpha\beta\beta\alpha\rangle - \frac{Y + J_S + J_L - 2J_a}{J_S + J_L - 2J_b}|\beta\alpha\alpha\beta\rangle \\ \psi_4 &= |\alpha\alpha\beta\beta\rangle + |\beta\beta\alpha\alpha\rangle - \frac{Y - 2J_a + 2J_b}{J_S + J_L - 2J_b}|\alpha\beta\alpha\beta\rangle - \frac{Y - 2J_a + 2J_b}{J_S + J_L - 2J_b}|\beta\alpha\beta\alpha\rangle + \\ &\quad + \frac{Y + J_S + J_L - 2J_a}{J_S + J_L - 2J_b}|\alpha\beta\beta\alpha\rangle + \frac{Y + J_S + J_L - 2J_a}{J_S + J_L - 2J_b}|\beta\alpha\alpha\beta\rangle \\ \psi_5 &= |\alpha\beta\beta\alpha\rangle - |\beta\alpha\alpha\beta\rangle \end{aligned} \tag{S9}$$

In order to demonstrate how HDVV Hamiltonian could be used as a correspondent to an effective Hamiltonian ($\hat{H}_{eff}$), if this system is to be appropriately modeled by Heisenberg Hamiltonian, we need to assemble $\hat{H}_{eff}$ for the problem and make one-to-one correspondence between $\hat{H}_{HDVV}$ and $\hat{H}_{eff}$. In this case, an appropriate matrix representation of the effective spin Hamiltonian is constructed from CASCI (4,4) wave functions because, as we see in Table S7, more than 99% of the wavefunction on this level of theory is composed of complete set of (4,4) neutral determinants that belong to $S_z = 0$ subspace. Hence, effective Hamiltonian theory can be used as a rigorous mathematical scheme to reduce the electronic Hamiltonian to a spin Hamiltonian of the HDVV

type by means of projection techniques.[32] We can build an effective Hamiltonian by the method of Bloch and orthogonalization approach of des Cloizeaux or Gram-Schmidt.[33–35]

The procedure of this approach starts by selecting the spin space (which is the model space) determining the low-lying energy spectrum. To construct the effective Hamiltonian, we need to project the approximate solutions of the exact nonrelativstic electronic Hamiltonian (in our case, (4,4) and (16,16) CASCI solutions) onto the N-dimensional model space S that contains the information of interest.[36] This formalism ensures one-to-one correspondence between the eigenvalues of the exact Hamiltonian and those of the effective Hamiltonian, and coincidence of the eigenfunctions of $\hat{H}_{eff}$ with the projection of the approximate solutions of the exact Hamiltonian onto the model space as closely as possible. Let us denote E_m and ϕ_m as energy and the corresponding eigenfunction of state m. It follows that:

$$\hat{H}\phi_m = E_m\phi_m \tag{S10}$$

We can further define a projector that targets the model space S as

$$\hat{P}_S = \sum_{I\in S}^{N} |I\rangle\langle I| \tag{S11}$$

Where $\{|I\rangle\}$ is an orthonormal basis of this model space, which in our case is 6 $S_z = 0$ determinants $|\alpha\alpha\beta\beta\rangle$, $|\beta\beta\alpha\alpha\rangle$, $|\alpha\beta\alpha\beta\rangle$, $|\beta\alpha\beta\alpha\rangle$, $|\alpha\beta\beta\alpha\rangle$, $|\beta\alpha\alpha\beta\rangle$ given in Table S7 which span $\hat{H}_{HDVV}$ shown in Eq. S5. In application, this requires previous localization step (note that our (4,4) and (16,16) CASCI were run with localized 4 frontier SOMOs). Hence, an effective Hamiltonian can be defined as given in Eq. S12:

$$\hat{H}_{eff}|\phi_{S,m}\rangle = E_m|\phi_{S,m}\rangle \tag{S12}$$

where $|\phi_{S,m}\rangle = \hat{P}_S|\phi_m\rangle$. Since the basis in which $\phi_{S,m}$ is expressed is not necessarily orthonormal, we use orthonormalized projections as proposed by des Cloizeaux given in Eq. S13 or orthonormalize this set using Gram-Schmidt procedure.

$$|\bar{\phi}_{S,m}\rangle = S^{-1/2}|\phi_{S,m}\rangle \tag{S13}$$

where $S_{m,q}$ are elements of the overlap matrix between states m and q as shown in Eq. S14:

$$S_{m,q} = \langle\phi_{S,m}\,|\,\phi_{S,q}\rangle \tag{S14}$$

Hence, finally, an effective Hamiltonian is obtained from its spectral decomposition, and its matrix representation in the model space spanned by, in our case, 6 $S_z = 0$ neutral determinants as given in Eq. S15.

$$H_{eff}(I,J) = \sum_{m=1}^{N}\langle I\,|\,\bar{\phi}_{S,m}\rangle E_m \langle\bar{\phi}_{S,m}\,|\,J\rangle \tag{S15}$$

Hence, the elements of $\hat{H}_{eff}$ can be directly compared to the elements of $\hat{H}_{HDVV}$, which in our case is given Eq. S5. By application of this procedure, we have computed the effective Hamiltonian for (4,4) CASCI results given in Eq. S16 and (16,16) CASCI results projected onto the (4,4) model space given in Eq. S17.

$$\hat{H}_{eff}((4,4)\text{ CASCI}) = \begin{pmatrix} 265.36 & 0.08 & -25.28 & -184.19 & -7.02 & -7.02 \\ 0.08 & 265.36 & -184.19 & -25.28 & -7.02 & -7.02 \\ -25.28 & -184.19 & 180.95 & 0.57 & -34.94 & -34.94 \\ -184.19 & -25.28 & 0.57 & 180.95 & -34.94 & -34.94 \\ -7.02 & -7.02 & -34.94 & -34.94 & 404.98 & 0.05 \\ -7.02 & -7.02 & -34.94 & -34.94 & 0.05 & 404.98 \end{pmatrix} \tag{S16}$$

$$\hat{H}_{eff}((16,16)\text{ CASCI}) = \begin{pmatrix} 226.47 & 0.06 & -21.67 & -156.03 & -6.46 & -6.46 \\ 0.06 & 226.47 & -156.03 & -21.67 & -6.46 & -6.46 \\ -21.67 & -156.03 & 153.26 & 0.42 & -29.96 & -29.96 \\ -156.03 & -21.67 & 0.42 & 153.26 & -29.96 & -29.96 \\ -6.46 & -6.46 & -29.96 & -29.96 & 344.27 & 0.03 \\ -6.46 & -6.46 & -29.96 & -29.96 & 0.03 & 344.27 \end{pmatrix} \tag{S17}$$

From comparing Eq. S6, Eq. S16 and Eq. S17, we see that it is trivial to determine value of each exchange-coupling constant from $\hat{H}_{HDVV}$. We see that largest exchange-coupling constant value is $J_S = 312.06\ \text{cm}^{-1}$ and the second largest is $J_a = 59.92\ \text{cm}^{-1}$, followed by $J_L = 43.34\ \text{cm}^{-1}$ and $J_b = 12.92\ \text{cm}^{-1}$. This would mean that both *subsystems* ***L*** and ***S*** have their electrons anti-ferromagnetically (spin anti-parallel) coupled and in turn this would mean that we would have a singlet ground state, which is indeed a case. Ovchinnikov's rule[37] predicts a singlet ground state for ***L***, ***S***, and ***LS***. The qualitative picture is similar for the effective Hamiltonian obtained from (4,4) and (16,16) CASCI calculations.

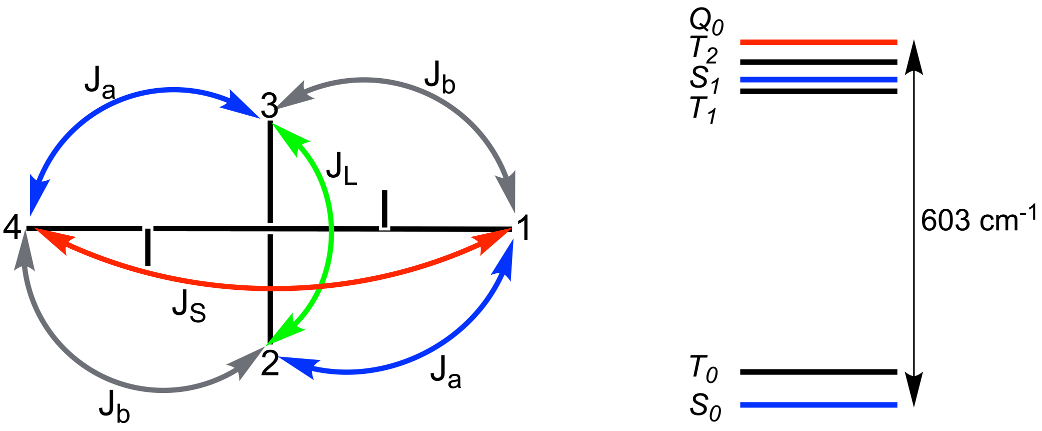

(a) **(b)**

Figure S6. (a) Scheme of strength of exchange-coupling interactions in the tetraradical(oid) ***LS***. Red is the strongest, blue is the second strongest closely followed by green and the weakest is gray. Rods sticking out from the horizontal line (1 – 4) correspond to the indole nitrogens. (b) low-energy spectrum of the tetraradical(oid) ***LS***.

As we see, the Heisenberg model gives us correct qualitative picture of what all CASSCF and CASCI calculations showed. Nonetheless, the quantitative picture of predicted by the Heisenberg model should be analyzed thoroughly. It is very important to point out that collective weight of the neutral determinants that span (4,4) $S_z = 0$ subspace for (16,16) CASCI is about 72% for all 6 magnetic spin states given in Table S8. This means that other determinants that lie out of (4,4) $S_z = 0$ and neutral determinants subspace have significant contribution (about 28%) into the wavefunction obtained from (16,16) CASCI calculation. Hence, even though qualitatively correct ground state is predicted, the wavefunction of this molecule cannot be fully spanned by (4,4) active space and consequent 6×6 model Hamiltonian $\hat{H}_{HDVV}$ built in the basis of six neutral determinants belonging to (4,4) active space. It is important to remark that our investigation through effective Hamiltonian reveals exchange-coupling interaction J_a between spin centers **1** and **2** (refer to Figure S4 for assignations) to be greater than between **2** and **3**, which is J_L. This can be partially rationalized from the resonance structures given in Figure S1. Electrons in centers **1** and **2** *could* pair-up antiferromagnetically and close the shell. Nonetheless, it would not be obvious to expect that J_a is greater than J_L. Hence, $\hat{H}_{HDVV}$ gives us additional insight into the dominant interactions in this magnetic system. This significant interaction J_a provides synergistic effect that keeps every open shell faithfully from diradical(oid)s ***L*** and ***S*** to create tetraradical(oid) ***LS***. These interactions can schematically be represented as shown in Figure S6a and low-energy spin spectrum is depicted in Figure S6b.

S4. Broken-symmetry UKS DFT and UHF benchmark results

This DFT and UHF benchmarks was performed in GAMESS version 2023 (R2).[18] Benchmark results for DFT PBE[38,39] functional hybrid with variable percentage of HF exchange (PBEX) are given in Table S9.

Table S9. DFT with variable HF % in PBE hybrid and UHF benchmark for all possible qualitatively distinct determinants for $S_z = 0, 1, 2$. Energies under each custom hybrid functional are given in atomic units. All quintets ($|\alpha\alpha\alpha\alpha\rangle$) have proper qualitative structure. Results with **closed-shell** qualitative structure are in **bold**, results with diradical(oid) structure are in ***bold italic***, and results with proper tetraradical(oid) qualitative structure are in *italic* fonts.

State	PBE_X_10	PBE_X_15	PBE_X_20	PBE_X_25	PBE_X_30	PBE_X_40	UHF
$\|\alpha\alpha\beta\beta\rangle$	**-2307.1915153914**	*-2307.2224897195*	*-2307.2422280590*	*-2307.2645556853*	*-2307.2894247925*	*-2307.3466601116*	*-2295.5812820174*
$\|\alpha\beta\alpha\beta\rangle$	**-2307.1915153914**	*-2307.1730022685*	**-2307.2109997783**	**-2307.2239721799**	**-2307.2390585887**	**-2307.2754653354**	*-2295.5190584744*
$\|\alpha\beta\beta\alpha\rangle$	*-2307.2054074073*	*-2307.2224897195*	*-2307.2422280590*	*-2307.2645556853*	*-2307.2894247925*	*-2307.3466601115*	*-2295.8028972884*
$\|\alpha\alpha\alpha\beta\rangle$	***-2307.1689286334***	***-2307.2146415387***	*-2307.2122121630*	***-2307.2487401370***	***-2307.2694702649***	***-2307.3181634094***	*-2295.5642976775*
$\|\alpha\alpha\beta\alpha\rangle$	*-2307.1864429577*	*-2307.1988761344*	***-2307.2280915415***	***-2307.2454355922***	***-2307.2650624302***	***-2307.3181634094***	*-2295.7804454967*
$\|\alpha\alpha\alpha\alpha\rangle$	-2307.2041453131	-2307.2214410763	-2307.2413144502	-2307.2637271118	-2307.2886465984	-2307.3459098382	-2295.8003577623

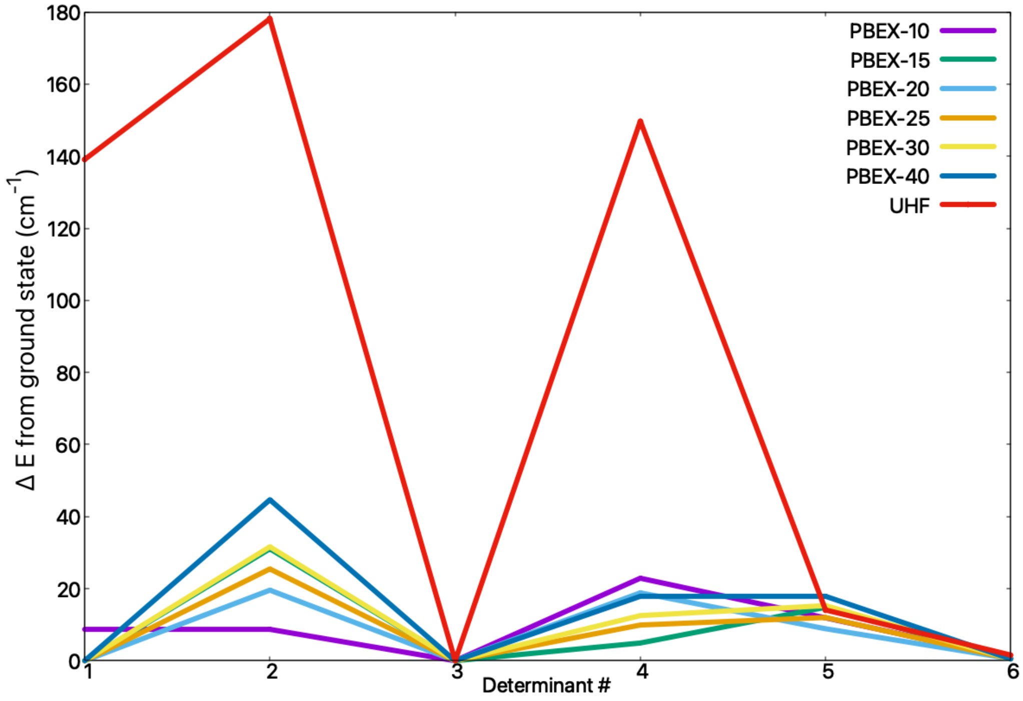

Figure S7. Benchmark of the Broken-symmetry calculations with different % of Hartree-Fock exchange in PBE-X hybrid functional and Unrestricted Hartree Fock. Determinant numbering is as follows $1 \equiv |\alpha\alpha\beta\beta\rangle$, $2 \equiv |\alpha\beta\alpha\beta\rangle$, $3 \equiv |\alpha\beta\beta\alpha\rangle$, $4 \equiv |\alpha\alpha\alpha\beta\rangle$, $5 \equiv |\alpha\alpha\beta\alpha\rangle$, $6 \equiv |\alpha\alpha\alpha\alpha\rangle$.

In order to properly visualize the variance of the results for particular spin states with different approximate Hamiltonian, we can look at the Figure S7. It is apparent that there is quite significant variability between energies of states and their relative distribution of minimum and maximum energies depending upon the % of HF exchange in their build. Also, there are significant differences between UHF and UKS results. Thus, this is an empirical demonstration that single-reference methods like HF and DFT are not sufficient to characterize all relevant spin states of ***LS***. It is noteworthy that some of the states calculated end up in a closed-shell solutions that does not describe the qualitative electronic structure of this molecule, which is indeed, according to all CASSCF and CASCI calculations, a tetraradical(oid). Hence, in description of ***LS***, we can rely only on CASCI and CASSCF results as qualitatively trustworthy. Nonetheless, it must be mentioned that quintet state is always described qualitatively correctly with respect to 4 frontier SOMOs that show its tetraradical(oid) identity. Hence, quintet DFT for a tetraradical(oid) can be partially relied upon for some basic qualitative properties, but only describing 4 SOMO part of the larger relevant active space as shown in Table S7 and Table S8. Furthermore, there are some spin states for which some functional properly captured the main aspects of the electronic structure. These cases can be used for the calculation of several properties of this tetraradical(oid).

S5. Study of Aromaticity of Tetraradical(oid) *LS*

Anisotropy of Induced Chemical Current (ACID)[40] calculations were done in order to explore the aromaticity of this tetraradical(oid). Since the qualitative electronic structure of several spin states modeled with PBE-X is aberrant, we need to select the combination of functional build and modeled spin states that reproduces correct qualitative structure of ***LS***. We could select PBE-X with 25% of HF exchange, which corresponds to PBE0 hybrid exchange-correlation functional.[41,42] For PBE0, the singlet ($|\alpha\alpha\beta\beta\rangle$) and quintet ($|\alpha\alpha\alpha\alpha\rangle$) show qualitatively correct tetraradical(oid) structure, as they manifest nearly 4 SONOs. Thus, exploring the aromaticity for these states with PBE0 is justified.

We can note that aromaticity can help explain the stability of the tetraradical(oid) electronic structure. Indeed, in the resonance structure in Figure S1, in which the molecule has 4 unpaired electrons (***LDSD***), all of its benzenoid rings possess 6 π electrons, making them Clar's aromatic sextets.[43] Other resonance structures (***LQSD***, ***LDSQ*** and ***LRSR***/***LRSR***′ in Figure S1) have only 2 Clar's π-sextets, making them less aromatic cumulatively. Moreover, these resonance structures **preclude** "*global aromaticity*" in the indolocarbazole aromatic backbone. The singlet and quintet states were calculated with PBE0/cc-pVDZ level of theory in Gaussian 09 program. From those results, ACID plots were computed separately and are given in Figure S8.

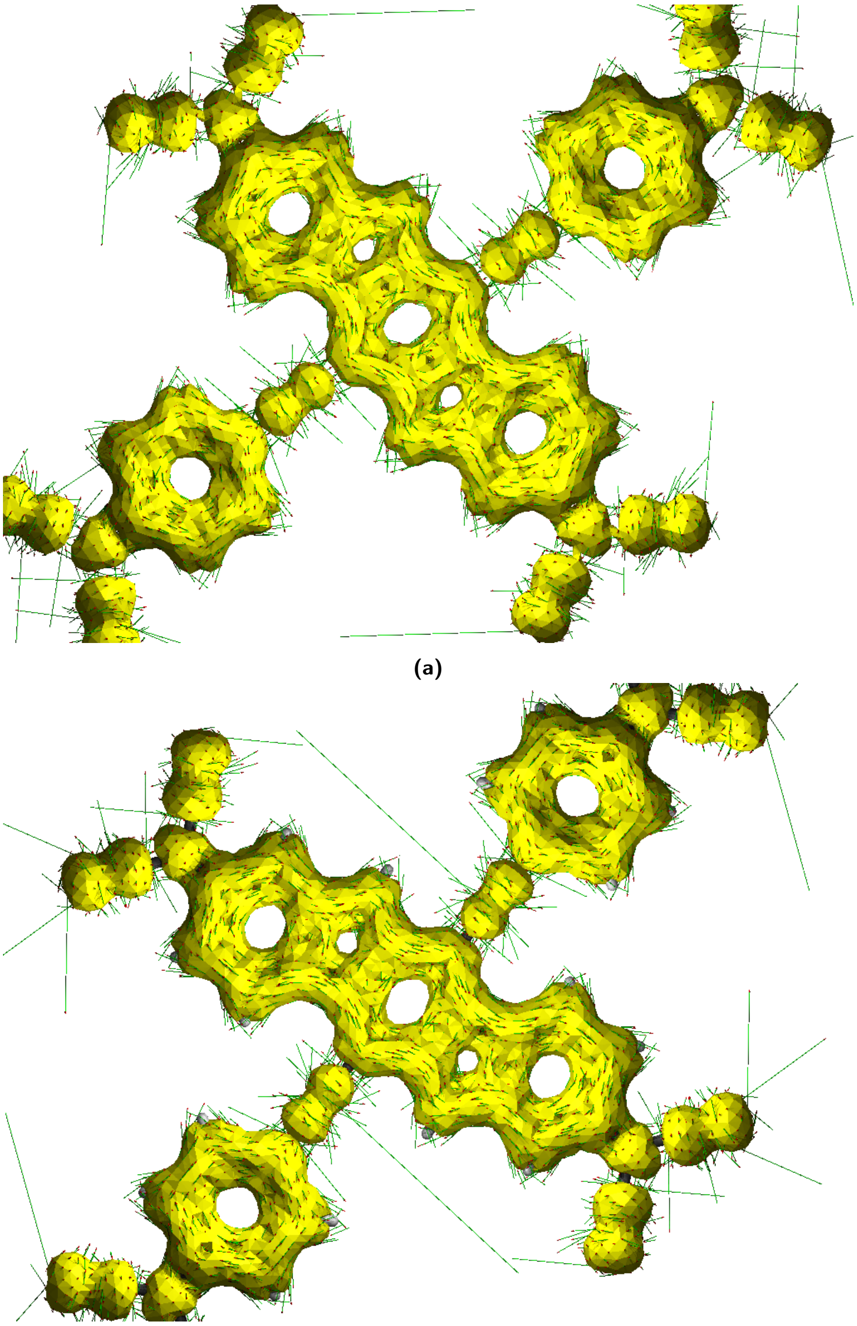

(a)

(b)

Figure S8. ACID plots for singlet (Figure S8a) and quintet (Figure S8b) states of ***LS*** calculated with PBE0/cc-pVDZ level of theory. Isosurface value is 0.030.

Since we see on the ACID plot that there is a continuous ring current across the indolocarbazole backbone, the compound possesses "*global aromaticity*" that is a further stabilizing factor than merely *local* aromaticity of Clar's π-sextets. Thus, in the resonance structure ***LDSD*** we have 5 Clar's π-sextets (three more than other resonance structures) and "*global aromaticity*" that stabilizes tetraradical(oid) electronic structure even more. From these results, we can conclude that the active space of this molecule contains two subsystems, which interact weakly but appreciably enough to affect the spin spectrum, because rest of the correlation is important for defining the spin states of a tetraradical(oid). The first of these subsystems is aromatic core(s) in the indolocarbazole and two other phenyl rings which act as an almost inner part of the active space, which appears in Table S8 as determinants of type S-L*, L-S*, S-S*, L-L*, S,S-S*,S*, L,L- L*,L*, L,S-L*,S*. These are excited determinants which contain excitations in the aromatic moieties of this molecule. This proves that the electron correlation in the aromatic part of the molecule is indeed important. The second of these subsystems is 4 SOMOs that define tetraradical(oid) with all possible spin states in low-energy spectrum within 603 cm^{-1}. It must be emphasized that conjugation between radical centers affects the spin spectrum of this tetraradical(oid), because the interaction/coupling between radical

centers is evidently non-zero. Nonetheless, this conjugation effect between radical centers is somewhat diminished by the presence of aromaticity of phenyl rings and "*global aromaticity*" of indolo[3,2-*b*]carbazole backbone. Hence, this shows that the nature of the tetraradical(oid), the role of the aromaticity in its stabilization and the degree of interaction between these aromatic and tetraradical subsystems define spin states of this fully planar π-conjugated tetraradical(oid) molecule with multiple thermally accessible singlet, triplet and quintet spin states.

In addition to the argument from the "*global aromaticity*" standpoint, it is also interesting to measure *local* aromaticity of the rings within ***LS***. For those, we use Multicenter Index (MCI) of aromaticity,[44,45] which was calculated for each ring given in Figure S9. For a given system, the MCI shows the simultaneous electron sharing between all centers, irrespective of the position of the atoms in the molecule.[45] MCI results for each ring of singlet, triplet and quintet states are given in Table S10. Notably, MCI values are hardly affected by changing the exchange-correlation functional, even by using CAM-B3LYP.

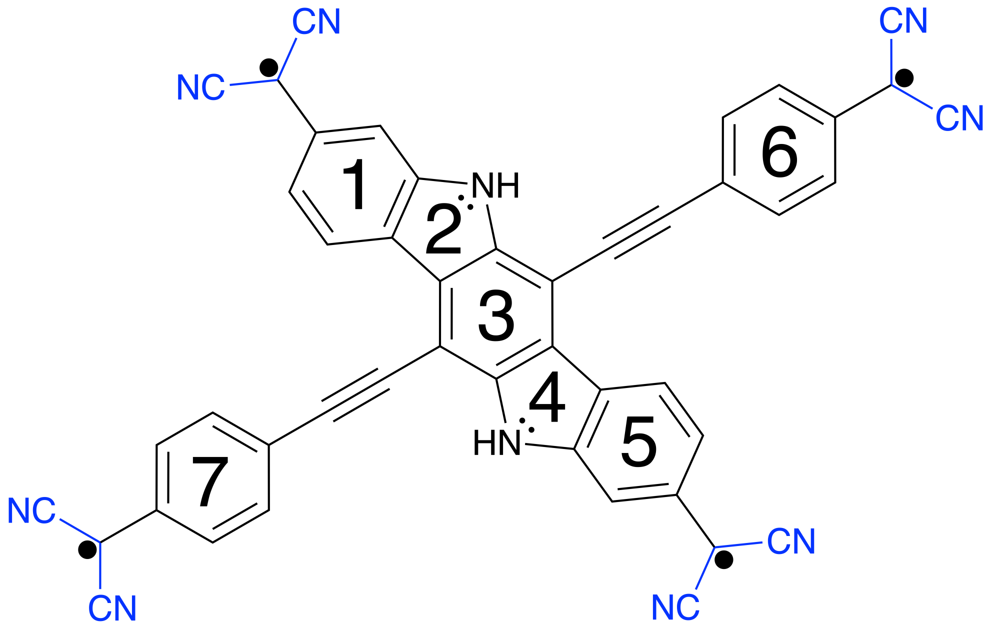

Figure S9. Ring numbering of ***LS***.

Table S10. Multicenter index (MCI) results for Singlet (S), Triplet (T), and Quintet (Q) tetraradical(oid) spin states of ***LS*** with different exchange-correlation functionals. Rings (R1, R2 ...) are numbered according to Figure S9.

Functional	State	Multicenter Index						
		R1	R2	R3	R4	R5	R6	R7
BLYP	S	0.028	0.014	0.014	0.014	0.028	0.036	0.036
	T	0.030	0.015	0.014	0.014	0.029	0.033	0.035
	Q	0.031	0.013	0.017	0.013	0.031	0.038	0.038
B3LYP	S	0.030	0.012	0.017	0.012	0.030	0.038	0.038
	T	0.032	0.013	0.018	0.012	0.031	0.037	0.038
	Q	0.032	0.012	0.019	0.012	0.032	0.039	0.039
CAM-B3LYP	S	0.032	0.011	0.021	0.011	0.032	0.039	0.039
	T	0.034	0.011	0.021	0.011	0.032	0.038	0.039
	Q	0.033	0.011	0.023	0.011	0.033	0.040	0.040
PBE0	S	0.031	0.012	0.018	0.012	0.031	0.038	0.038
	T	0.032	0.012	0.018	0.012	0.031	0.037	0.038
	Q	0.032	0.012	0.020	0.012	0.032	0.039	0.039

These calculations were performed in AMS (with ADF version 2023.104). To obtain these states, we start with

a quintet state calculation. For both states, we use the quintet converged guess orbitals, and flip the spin on two of the radical-bearing atoms (using "SpinFlip" keyword in ADF engine) to obtain a singlet state, while for a triplet state, we flip the spin on one of the radical-bearing atoms. All of these converged states were verified to qualitatively approximate the tetraradical(oid) electronic structure. These results show that tetraradical(oid) spin states of ***LS*** involve aromatic rings as depicted in the ***LDSD*** resonance structure in Figure S1. The values of MCI given in Table S10 are close to MCI values of the benzenoid rings in other aromatic compounds.[44,45]

S6. Study of Tetraradical(oid) *LSH*

Since cyano groups are somewhat stabilizing factor for radical centers via π-conjugation, it is pertinent to study the molecule which has hydrogens instead of cyano groups as terminal substituents in ***LS***. We will refer to this molecule as ***LSH***, for which resonance structures are given in Figure S10. Geometry optimization, CASSCF(4,4),

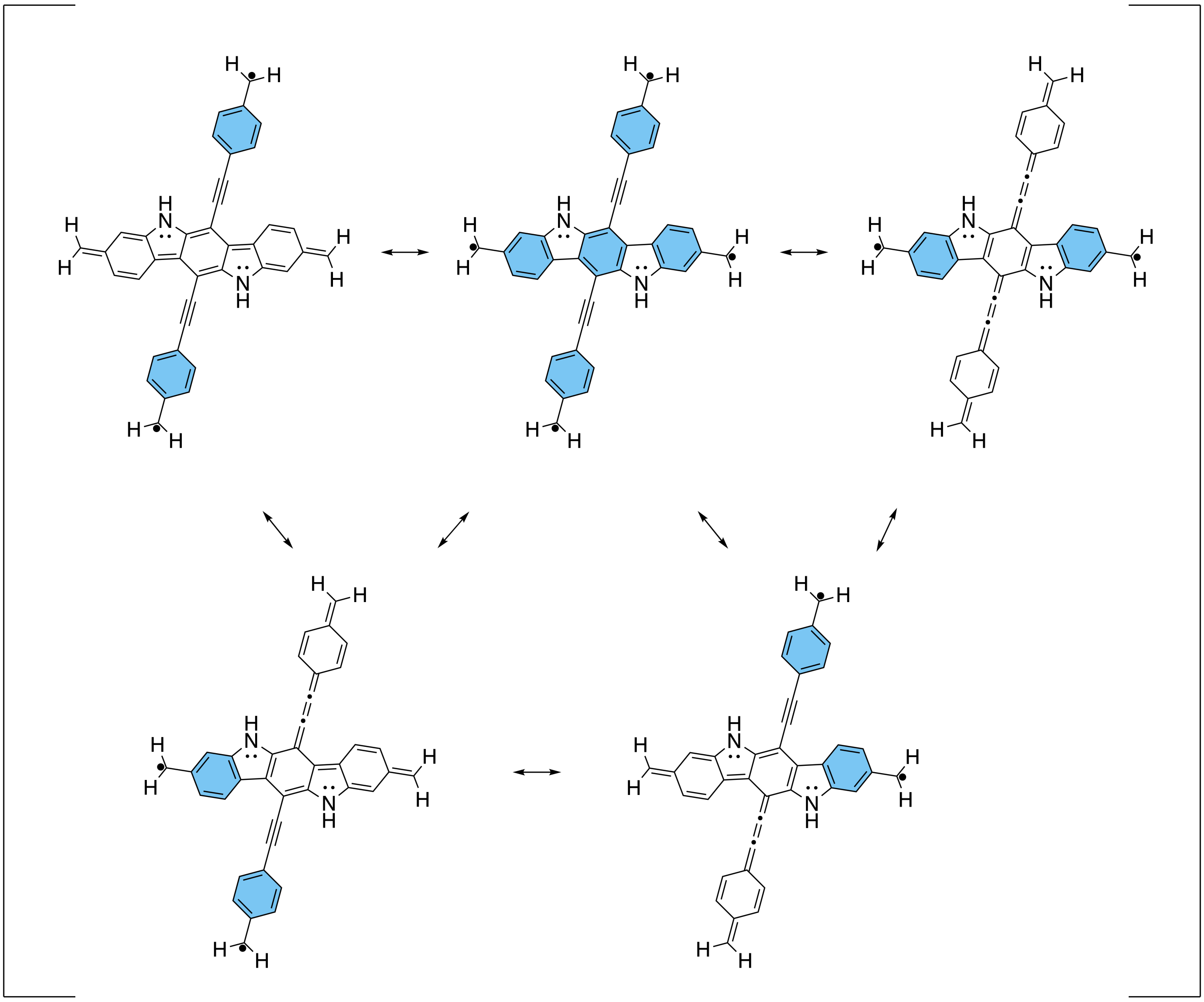

Figure S10. Resonance structures of 6,12-(bis((4-methylene-phenyl)ethynyl))-3,9-dimethylene-indolo[3,2-*b*]carbazole referred to as ***LSH***.

CASSCF(16,16), CASCI(4,4) and CASCI(16,16) calculations were run for ***LSH*** with exactly the same procedure and level of theory as for ***LS***. Results of CASSCF(4,4) calculations are given in Table S11 and results of CASSCF(16,16) calculations are given in Table S12. Since ***LS*** and ***LSH*** overlap in most of their structures, it turns out it is possible to make same assignations to frontier NOs of ***LSH*** as it was for ***LS***. Hence, we can have a common assignation of frontier NOs as given in Figure S5 and we can analyze spin states of ***LSH*** accordingly. From CASSCF(4,4) and CASSCF(16,16) results for ***LS*** and ***LSH***, we can see that ***LSH*** has its diradical and tetraradical character close to and slightly lower than those of ***LS***. Thus, since we have previously shown that ***LS*** is a persistent tetraradical(oid), we can state the same about ***LSH***. This comparison shows that even though cyano groups stabilize radical centers, contribution of this effect in overall stabilization of the tetraradical electronic structure is not determinative. The determining factors for the stability of tetraradical electronic structure of ***LS*** and ***LSH*** are cross-conjugated π-topology and aromaticity. To explore the wavefunction of states of ***LSH***,

CASCI(4,4) and CASCI(16,16) were run from CASSCF(16,16) Q_0 converged state with (4,4) frontier localized subspace (see the orbital numbering assignations in Figure S4). CASCI(4,4) results are given in Table S13 and CASCI(16,16) results are given in Table S14.

Table S11. CASSCF(4,4) states of ***LSH*** determined by state-specific calculations. For all the calculations, UHF quintet NOs were used as initial guess. Under HONO, LUNO, etc. three columns represent symmetry of the particular orbital, its symbolic representation according to Figure S5 and NO occupation number.

State	Symmetry	Energy (a.u.)	CASSCF NOs symmetry, identity and occupation number												ΔE from G. S. (cm^{-1})
			HONO − 1			HONO			LUNO			LUNO + 1			
S_0	A_g	-1561.7656766156	A_u	S_+	1.142	A_u	L_+	1.036	B_g	L_-	0.962	B_g	S_-	0.860	0.00
T_0	B_u	-1561.7655429785	A_u	S_+	1.142	B_g	L_-	1.001	A_u	L_+	0.998	B_g	S_-	0.860	29.33
S_1	A_g	-1561.7644852268	A_u	C_0	1.049	B_g	C_1	1.006	B_g	C_1'	0.987	A_u	C_2	0.959	261.48
T_1	B_u	-1561.7644370946	A_u	L_+	1.045	B_g	S_-	0.998	A_u	S_+	0.997	B_g	L_-	0.959	272.04
T_2	A_g	-1561.7644279814	A_u	C_0	1.034	B_g	C_1	1.017	B_g	C_1'	0.983	A_u	C_2	0.860	274.04
Q_0	A_g	-1561.7642517325	A_u	D_0 [a]	1.000	B_g	D_1	1.000	A_u	D_2	1.000	B_g	D_1'	1.000	312.73

[a] D_0, D_1, D_1', D_2 means **distorted** orbitals. Overall electron density corresponds to the same as if they were C_0, C_1, C_1', C_2 orbitals.

Table S12. CASSCF(16,16) states of ***LSH*** determined by state-specific calculations. For all the calculations, UHF quintet NOs were used as initial guess. Under HONO, LUNO, etc. three columns represent symmetry of the particular orbital, its symbolic representation according to Figure S5 and NO occupation number.

State	Symmetry	Energy (a.u.)	CASSCF NOs symmetry, identity and occupation number												ΔE from G. S. (cm^{-1})
			HONO − 1			HONO			LUNO			LUNO + 1			
S_0	A_g	-1561.9113563163	A_u	S_+	1.263	A_u	L_+	1.049	B_g	L_-	0.948	B_g	S_-	0.742	0.00
T_0	B_u	-1561.9108982411	A_u	S_+	1.264	B_g	L_-	1.001	A_u	L_+	0.997	B_g	S_-	0.740	100.54
S_1	A_g	-1561.9074478913	A_u	C_0	1.081	B_g	C_1	1.010	B_g	C_1'	0.976	A_u	C_2	0.936	857.80
T_1	B_u	-1561.9074101414	A_u	D_0	1.074	A_u	D_2	0.997	B_g	S_-	0.994	B_g	L_-	0.937	866.09
T_2	A_g	-1561.9073234317	A_u	C_0	1.060	B_g	C_1	1.026	B_g	C_1'	0.971	A_u	C_1	0.945	885.12
Q_0	A_g	-1561.9068303423	A_u	C_0	1.005	A_u	C_2	1.000	B_g	D_1 [a]	1.000	B_g	D_1'	0.945	993.34

[a] D_0, D_1, D_1', D_2 means **distorted** orbitals. Overall electron density corresponds to the same as if they were C_0, C_1, C_1', C_2 orbitals.

Table S13. CASCI(4,4) of ***LSH*** from CASSCF(16,16) quintet converged orbitals as initial guess. Under HONO, LUNO, etc. three columns represent symmetry of the particular orbital, its symbolic representation according to Figure S5 and NO occupation number.

State	Symmetry	Energy (a.u.)	CASCI NOs occupancy, identity and symmetry												ΔE from G. S. (cm^{-1})
			HONO − 1			HONO			LUNO			LUNO + 1			
S_0	A	-1561.7325502111	A	S_+	1.221	A	L_+	1.056	A	L_-	0.936	A	S_-	0.784	0.00
T_0	A	-1561.7320621125	A	S_+	1.215	A	L_-	1.002	A	L_+	0.993	A	S_-	0.789	107.13
S_1	A	-1561.7295650149	A	C_0	1.101	A	C_1	1.018	A	C_1'	0.969	A	C_2	0.912	655.17
T_1	A	-1561.7295093177	A	L_+	1.094	A	S_-	0.996	A	S_+	0.993	A	L_-	0.916	667.40
T_2	A	-1561.7294239764	A	C_0	1.074	A	C_1	1.035	A	C_1'	0.965	A	C_2	0.926	686.13
Q_0	A	-1561.7288601300	A	S_+	1.000	A	L_-	1.000	A	L_+	1.000	A	S_-	1.000	809.88

Table S14. CASCI(16,16) of ***LSH*** from CASSCF(16,16) quintet converged orbitals as initial guess. Under HONO, LUNO, etc. three columns represent symmetry of the particular orbital, its symbolic representation according to Figure S5 and NO occupation number.

State	Symmetry	Energy (a.u.)	CASCI NOs occupancy, identity and symmetry												ΔE from G. S. (cm^{-1})
			HONO − 1			HONO			LUNO			LUNO + 1			
S_0	A_g	-1561.9098930030	A	S_+	1.167	A	L_+	1.040	A	L_-	0.962	A	S_-	0.860	0.00
T_0	B_u	-1561.9094819235	A	S_+	1.163	A	L_-	1.001	A	L_+	0.998	A	S_-	0.860	90.22
S_1	A_g	-1561.9073791723	A	C_0	1.074	A	C_1	1.011	A	C_1'	0.987	A	C_2	0.959	551.72
T_1	B_u	-1561.9073575888	A	L_+	1.067	A	S_+	0.996	A	S_-	0.997	A	L_-	0.959	556.46
T_2	A_g	-1561.9072775028	A	C_0	1.056	A	C_1	1.024	A	C_1	0.983	A	C_2	0.860	574.04
Q_0	A_g	-1561.9068303187	A	C_0	1.005	A	C_2	1.000	A	D_1 [a]	1.000	A	D_1'	1.000	672.18

[a] D_0, D_1, D_1', D_2 means **distorted** orbitals. Overall electron density corresponds to the same as if they were C_0, C_1, C_1', C_2 orbitals.

We can also determine exchange-coupling constants between the radical centers with exactly the same procedure as we did for ***LS***, as explained in Section S3.2. Since the procedure of CASSCF and CASCI calculations have been the same including the ordering of localized frontier orbitals from which CASCI calculations were run, we

Table S15. CASCI(4,4) determinant contributions for 6 near-degenerate tetraradical(oid) spin states of ***LSH***. These states are obtained for $S_z = 0$ basis from CASSCF(16,16) converged quintet localized (in (4,4) SOMOs subspace) initial guess orbitals.

	S_0	T_0	S_1	T_1	T_2	Q_0
ΔE from ground state (cm^{-1})	0.00	107.13	655.17	667.40	686.13	809.88
$\lvert\alpha\alpha\beta\beta\rangle$	0.4228316954	0.4186802024	-0.3887336637	0.5667939039	0.0000000000	0.4082482905
$\lvert\beta\beta\alpha\alpha\rangle$	0.4228316954	-0.4186802024	-0.3887336637	-0.5667939039	0.0000000000	0.4082482905
$\lvert\alpha\beta\alpha\beta\rangle$	0.5462292751	-0.5639631369	0.1737876524	0.4208062212	0.0000000000	-0.4082482905
$\lvert\beta\alpha\beta\alpha\rangle$	0.5462292751	0.5639631369	0.1737876524	-0.4208062212	0.0000000000	-0.4082482905
$\lvert\alpha\beta\beta\alpha\rangle$	0.1233975797	0.0000000000	0.5625213162	0.0000000000	-0.7059126895	0.40824829055
$\lvert\beta\alpha\alpha\beta\rangle$	0.1233975797	0.0000000000	0.5625213162	0.0000000000	0.7059126895	0.4082482905
Sum of coefficients $\sum_{I\in CAS} c_I^2$	0.9848	0.9867	0.9955	0.9967	0.9966	1.0000

Table S16. CASCI(16,16) determinant contributions for 6 near-degenerate tetraradical(oid) spin states of ***LSH***. These states are obtained for $S_z = 0$ basis from CASSCF(16,16) converged quintet localized (in (4,4) SOMOs subspace) initial guess orbitals.

	S_0	T_0	S_1	T_1	T_2	Q_0
ΔE from ground state (cm^{-1})	0.00	90.22	551.72	556.46	574.04	672.18
$\lvert 222222\alpha\alpha\beta\beta000000\rangle$	0.3519457136	0.3476166619	-0.3245076566	-0.4725669194	0.0000000000	0.3391042030
$\lvert 222222\beta\beta\alpha\alpha000000\rangle$	0.3519457140	-0.3476166608	-0.3245076022	0.4725669575	0.0000000000	0.3391042026
$\lvert 222222\alpha\beta\alpha\beta000000\rangle$	0.4565641304	-0.4717555310	0.1430559821	-0.3483053567	0.0000000000	-0.3391034914
$\lvert 222222\beta\alpha\beta\alpha000000\rangle$	0.4565641293	0.4717555314	0.1430560222	0.3483053399	0.0000000000	-0.33910349265
$\lvert 222222\alpha\beta\beta\alpha000000\rangle$	0.1046183230	0.0000000000	0.4675673034	0.0000000000	-0.5869842187	0.3391013946
$\lvert 222222\beta\alpha\alpha\beta000000\rangle$	0.1046183230	0.0000000000	0.4675672949	0.0000000000	0.5869842230	0.3391013987
Sum of coefficients $\sum_{I\in CAS(4,4)} c_I^2$	0.6865	0.6868	0.6888	0.6893	0.6891	0.6899

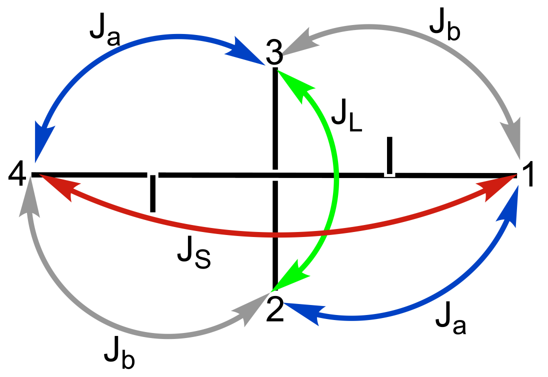

(a)

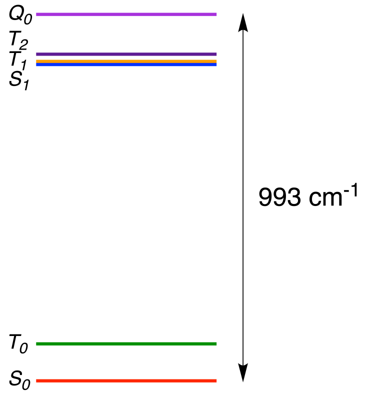

(b)

Figure S11. (a) Scheme of strength of exchange-coupling interactions in the tetraradical(oid) ***LSH***. Dark red is the strongest by more than factor of 4 relative to dark blue that is the second strongest, followed by moderate green and the weakest is gray. Rods sticking out from the horizontal line (1 – 4) correspond to the indole nitrogens. (b) low-energy spectrum of the tetraradical(oid) ***LSH***.

can use the same model Hamiltonian for ***LSH*** as we did for ***LS***. Determinant contributions from CASCI(4,4) and CASCI(16,16) results for ***LSH*** are given in Table S15 and Table S16, respectively. If we compare composition of the wavefunction for each state for ***LSH***, they parallel those of ***LS*** for the corresponding states very well. From CASCI(4,4) results, we find that $J_{12} = J_{34} = J_a = 141.26$ cm^{-1}, $J_{13} = J_{24} = J_b = 17.86$ cm^{-1}, $J_{23} = J_L = 90.74$ cm^{-1}, $J_{14} = J_S = 627.02$ cm^{-1}. From CASCI(16,16) results, we find that $J_{12} = J_{34} = J_a = 116.92$ cm^{-1}, $J_{13} = J_{24} = J_b = 19.02$ cm^{-1}, $J_{23} = J_L = 75.56$ cm^{-1}, $J_{14} = J_S = 521.08$ cm^{-1}. The ordering of strengths of these exchange-coupling constants is same for ***LSH*** as for ***LS***. Hence, the scheme for ***LSH*** would resemble the scheme of ***LS*** as shown in Figure S11a with moderated color shades to emphasize difference from ***LS***. The low-energy spin spectrum is for ***LSH*** is given in Figure S11b. We find that the range of energy of states in low-energy spectrum increases by about 390 cm^{-1} and exchange-couplings are also stronger in ***LSH*** compared to ***LS***. Nonetheless, even though cyano substituents increase the tetraradical character slightly and decrease the range of energy of states in low-energy spectrum, their effect on the electronic structure of the tetraradical(oid) is rather small. Therefore, the tetraradical(oid) electronic structure of both ***LS*** and ***LSH*** is stabilized by cross-conjugation and aromaticity.

S7. Study of Diradical(oid)s *L* and *S*

In order to gain further insight in the properties of this tetraradical(oid), it could be useful to analyze its conceptual constituent diradical(oid)s. The structure for diradical(oid) ***L*** is given in Figure S12 and the structure of ***S*** is given in Figure S13. Their geometries have been optimized with BLYP/TZP level of theory in ADF in the triplet state because of their diradical(oid) nature. Note that ***L*** has D_{2h} point group symmetry and was optimized as such, and ***S*** has C_{2h} symmetry and was optimized accordingly as well.

CASSCF(14,14) and CASCI(14,14) (or CASCI(16,16)) calculations were run for each diradical(oid) molecule. All calculations were initiated from UHF triplet NOs guess as it is one of the best initial guess for diradical systems.[27] The results for ***S*** are given in Table S17 for CASSCF(14,14) and in Table S18 for CASCI(14,14). As we see, we have two spin states, S_0 with A_g symmetry as a ground state and T_0 with B_u symmetry as a first excited state with singlet-triplet gap ($\Delta E_{S-T} = E_T - E_S$) amounting to only 1.63 kcal/mol according to CASSCF(14,14) and 0.77 kcal/mol according to CASCI(14,14). Higher excited states are too distant from these two states by more than 75 kcal/mol for each case. Hence, this molecule ***S*** is a persistent diradical(oid).

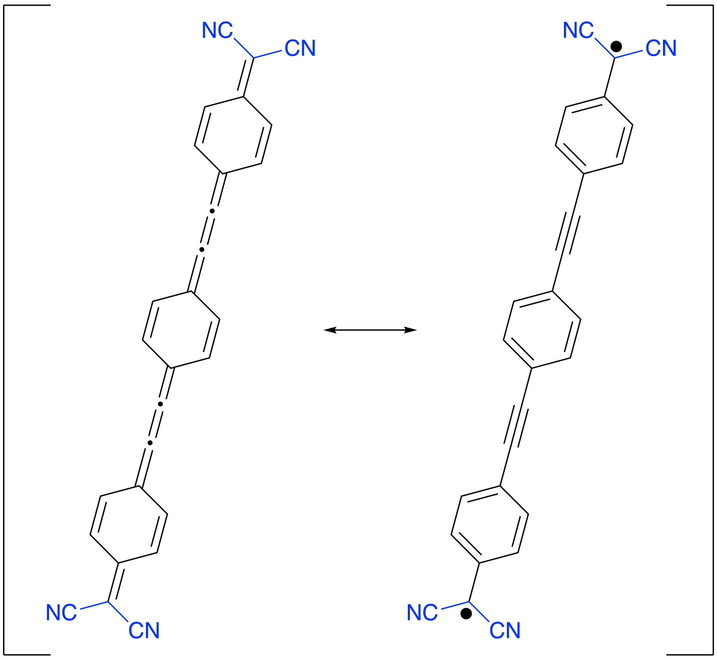

Figure S12. Resonance structures of 2,2′-((1,4-phenylenebis(ethyne-2,1-diyl))bis(4,1-phenylene))dimalononitrile referred to as ***L***.

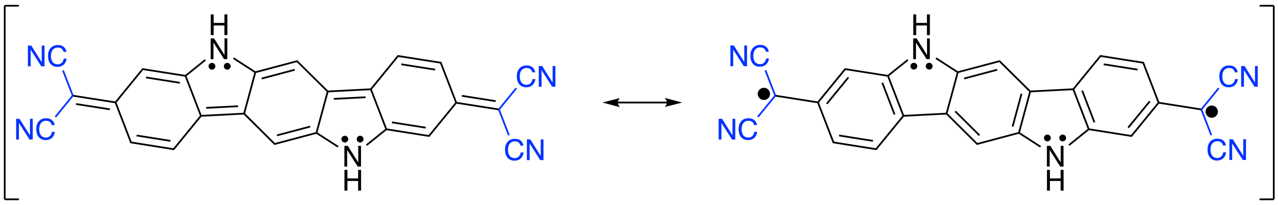

Figure S13. Resonance structures of the 2,2′-(5,11-dihydroindolo[3,2-*b*]carbazole-3,9-diyl)dimalononitrile referred to as ***S***.

Table S17. CASSCF(14,14) results results for ***S*** from UHF triplet NOs as initial guess.

									ΔE from G. S. ($kcal \cdot mol^{-1}$)
CASSCF NOs symmetry, identity and occupation number									
State	Symmetry	Energy (a.u.)	HONO			LUNO			ΔE from G. S. ($kcal \cdot mol^{-1}$)
S_0	A_g	-1241.5385394676	A_u	S_+	1.205	B_g	S_-	0.801	0.00
T_0	B_u	-1241.5359426290	A_u	S_-	1.006	B_g	S_-	0.999	1.63

The results for molecule ***L*** are given in Table S19 for CASCI(16,16) calculations and in Table S20 for CASSCF(14,14). We find that ***L*** has two very close-lying spin states T_0 with B_{3u} symmetry and S_0 with A_g symmetry with singlet-

Table S18. CASCI(14,14) calculations results for ***S*** from UHF triplet NOs as initial guess.

CASCI NOs symmetry, identity and occupation number									
State	Symmetry	Energy (a.u.)	HONO			LUNO			ΔE from G. S. ($kcal \cdot mol^{-1}$)
S_0	A_g	-1241.4829022983	A_u	S_+	1.119	B_g	S_-	0.881	0.00
T_0	B_u	-1241.4816741649	A_u	S_+	1.003	B_g	S_-	0.997	0.77
S_1	A_g	-1241.3617244293	A_u	S_+	1.381	B_g	S_-	1.054	76.04
T_1	A_g	-1241.3582580005	A_u	S_+	1.415	B_g	S_-	1.190	78.22
S_2	B_u	-1241.3560633462	A_u	S_+	1.312	B_g	S_-	1.305	79.59
T_2	B_u	-1241.3553562960	B_g	S_-	1.304	A_u	S_+	1.000	80.04

Table S19. CASCI(16,16) calculations results for ***L*** from UHF triplet NOs as initial guess.

CASCI NOs symmetry, identity and occupation number									
State	Symmetry	Energy (a.u.)	HONO			LUNO			ΔE from G. S. ($kcal \cdot mol^{-1}$)
T_0	B_{3u}	-1285.0990513955	B_{1u}	L_+	1.001	B_{2g}	L_-	0.999	0.00
S_0	A_g	-1285.0970870142	B_{1u}	L_+	1.044	B_{2g}	L_-	0.956	1.23
S_1	A_g	-1284.9922337944	B_{1u}	L_+	1.284	B_{2g}	$L-$	1.074	67.03
S_2	B_{3u}	-1284.9915983467	B_{2g}	L_-	1.196	B_g	L_+	1.188	67.43
T_1	A_g	-1284.9908543531	B_{1u}	L_+	1.245	B_{2g}	L_-	1.146	67.89
T_2	B_{3u}	-1284.9905198538	B_{2g}	L_-	1.195	B_{1u}	L_+	1.184	68.10

Table S20. CASSCF(14,14) calculations results for ***L*** from UHF triplet NOs as initial guess.

CASSCF NOs symmetry, identity and occupation number									
State	Symmetry	Energy (a.u.)	HONO			LUNO			ΔE from G. S. ($kcal \cdot mol^{-1}$)
S_0	A_g	-1285.1483119564	A_u	L_+	1.047	B_g	L_-	0.958	0.00
T_0	B_{3u}	-1285.1481335160	A_u	L_+	1.003	B_g	L_-	1.001	0.11
S_1	A_g	-1285.0499069625	A_u	L_+	1.240	B_g	L_-	1.037	61.75
T_1	A_g	-1285.0485091253	A_u	L_+	1.201	B_g	L_-	1.105	62.63
S_2	B_{3u}	-1285.0478791136	B_g	L_-	1.158	A_u	L_+	1.148	63.02
T_2	B_{3u}	-1285.0477919517	B_g	L_-	1.148	A_u	L_+	1.144	63.08

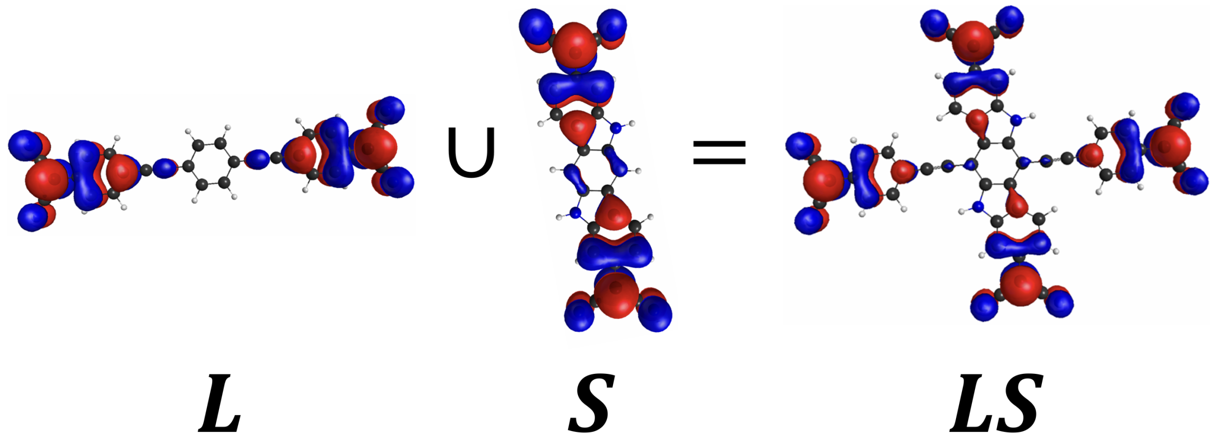

Figure S14. Representation of conceptual union (∪) of two diradical(oid) molecules ***L*** and ***S*** producing ***LS*** tetraradical(oid) molecule.

triplet gap of (ΔE_{S-T}) -1.23 $kcal \cdot mol^{-1}$ according to CASCI(16,16) calculations and 0.11 $kcal \cdot mol^{-1}$ according to CASSCF(14,14) calculations. As we see, both molecules ***S*** and ***L*** have large diradical character. Furthermore, we saw that their conceptual union through central benzene ring of each produces tetraradical(oid) with accessible paramagnetic states. To explain this conclusion pictorially, we can refer to Figure S14. Therefore, in this work,

we described two subsystems $\boldsymbol{S}$ and $\boldsymbol{L}$, which are independently diradical(oid)s and through their conceptual union we obtained tetraradical(oid) $\boldsymbol{LS}$, which has 6 spin states in the range of 603 cm^{-1}. The merge of these diradical(oid)s is synergistic towards maintaining unpaired electrons to form a tetraradical(oid) as reflected by significant cross-couplings between the diradical(oid) subunits.

S8. Optimized Geometries

The optimized geometry of tetraradical(oid) $\boldsymbol{LS}$ with BLYP/TZP quintet state is given below. This molecule has C_{2h} point group symmetry and has been optimized as such. As mentioned in the methodology, planar geometry results irrespective of whether the starting geometry is planar or not.
Total bonding energy: -18.797140365861537 $a.u.$

```
72
C46H16N10 optimized with ADF in AMS.
C        0.00643515340622      3.37826795057296       0.00000000000000
C       -1.18560529424071      2.57882443460124       0.00000000000000
N        1.10403774321341      2.52000057391310       0.00000000000000
C       -0.02119778300087      4.76643873224609       0.00000000000000
C       -2.43874319043890      3.23440236096129       0.00000000000000
C       -2.48643021633779      4.61864097041178       0.00000000000000
C       -1.28935600546168      5.40996808715648       0.00000000000000
N        3.65333809292614     -8.07236475824837       0.00000000000000
H        0.89511505605194      5.35373241224626       0.00000000000000
H        3.35865746736626     -2.65652471693477       0.00000000000000
C       -0.76633233490131      1.19854257669916       0.00000000000000
H       -3.35865746736626      2.65652471693477       0.00000000000000
H       -3.44847050269608      5.12596325407791       0.00000000000000
C        0.66913935530420      1.20607253815175       0.00000000000000
C        1.47934342554760      0.03870546118421       0.00000000000000
C        0.76633233490131     -1.19854257669916       0.00000000000000
C       -0.66913935530420     -1.20607253815175       0.00000000000000
C       -1.47934342554760     -0.03870546118421       0.00000000000000
H        2.07919665413033      2.79861953056489       0.00000000000000
H       -2.07919665413033     -2.79861953056489       0.00000000000000
C        1.18560529424071     -2.57882443460124       0.00000000000000
C       -0.00643515340622     -3.37826795057296       0.00000000000000
N       -1.10403774321341     -2.52000057391310       0.00000000000000
N       -0.78811861366206     -8.28522067104072       0.00000000000000
C        2.43874319043890     -3.23440236096129       0.00000000000000
C        2.48643021633779     -4.61864097041178       0.00000000000000
C        1.28935600546168     -5.40996808715648       0.00000000000000
C        0.02119778300087     -4.76643873224609       0.00000000000000
N        0.78811861366206      8.28522067104072       0.00000000000000
H        3.44847050269608     -5.12596325407791       0.00000000000000
H       -0.89511505605194     -5.35373241224626       0.00000000000000
C       -1.36934765675879      6.85479158838621       0.00000000000000
C       -2.61478004940291      7.53062967607841       0.00000000000000
C       -0.19926812840179      7.65512139091326       0.00000000000000
N       -3.65333809292614      8.07236475824837       0.00000000000000
C        1.36934765675879     -6.85479158838621       0.00000000000000
C        2.61478004940291     -7.53062967607841       0.00000000000000
C        0.19926812840179     -7.65512139091326       0.00000000000000
C       -2.88307426422434     -0.14747224696756       0.00000000000000
C        2.88307426422434      0.14747224696756       0.00000000000000
C       -4.09927292419589     -0.31415481077973       0.00000000000000
C        4.09927292419589      0.31415481077973       0.00000000000000
C        9.76298495594650      0.97924846531083       0.00000000000000
H        8.38971672916676     -1.36748417535800       0.00000000000000
H        5.94582568588618     -1.65253827352582       0.00000000000000
```

```
H        7.88381635680226       2.94335051091979       0.00000000000000
H       -5.94582568588618       1.65253827352582       0.00000000000000
C        5.50111735797724       0.47820741268003       0.00000000000000
C        6.08656390708858       1.77865265831601       0.00000000000000
C        7.45945354728187       1.94112308528297       0.00000000000000
C        8.33168927505058       0.81076947984367       0.00000000000000
C        7.74473466008886      -0.49079571972791       0.00000000000000
C        6.37152550317442      -0.65153728196386       0.00000000000000
H        5.44043286186097       2.65413544621793       0.00000000000000
C       -5.50111735797724      -0.47820741268003       0.00000000000000
C       -6.08656390708858      -1.77865265831601       0.00000000000000
C       -7.45945354728187      -1.94112308528297       0.00000000000000
C       -8.33168927505058      -0.81076947984367       0.00000000000000
C       -7.74473466008886       0.49079571972791       0.00000000000000
C       -6.37152550317442       0.65153728196386       0.00000000000000
H       -5.44043286186097      -2.65413544621793       0.00000000000000
H       -7.88381635680226      -2.94335051091979       0.00000000000000
C       -9.76298495594650      -0.97924846531083       0.00000000000000
H       -8.38971672916676       1.36748417535800       0.00000000000000
C       10.35502509780306       2.26799167517209       0.00000000000000
N       10.82062845254428       3.34252708499749       0.00000000000000
C       10.63912718236024      -0.13588842768511       0.00000000000000
N       11.34413648533630      -1.07096548660261       0.00000000000000
C      -10.63912718236024       0.13588842768511       0.00000000000000
N      -11.34413648533630       1.07096548660261       0.00000000000000
C      -10.35502509780306      -2.26799167517209       0.00000000000000
N      -10.82062845254428      -3.34252708499749       0.00000000000000
```

The quintet-optimized C_{2h} geometry of molecule ***LSH*** is given below (BLYP/TZP level of theory).
Total bonding energy: -15.227968755236777 $a.u.$

```
64
C38H24N2 optimized with ADF in AMS.
C        0.01011874753548       3.38482395475182       0.00000000000000
C       -1.18232260055731       2.58469159445773       0.00000000000000
N        1.10587793745895       2.52151055403438       0.00000000000000
C       -0.01704368746808       4.77349612328418       0.00000000000000
C       -2.43651358036049       3.24389281365683       0.00000000000000
C       -2.48327238950320       4.62653095086246       0.00000000000000
C       -1.28697975285501       5.43955724180865       0.00000000000000
H       10.19687917500584       1.96541785565724       0.00000000000000
H        0.90212820819461       5.35907918165227       0.00000000000000
H        3.35526809822659      -2.66261056387841       0.00000000000000
C       -0.76519335980291       1.20157040692758       0.00000000000000
H       -3.35526809822659       2.66261056387841       0.00000000000000
H       -3.44670109349714       5.13513506649372       0.00000000000000
C        0.66744533563127       1.20412154120484       0.00000000000000
C        1.47684952835503       0.03743856312109       0.00000000000000
C        0.76519335980291      -1.20157040692758       0.00000000000000
C       -0.66744533563127      -1.20412154120484       0.00000000000000
C       -1.47684952835503      -0.03743856312109       0.00000000000000
H        2.08222856762629       2.79290125273512       0.00000000000000
H       -2.08222856762629      -2.79290125273512       0.00000000000000
C        1.18232260055731      -2.58469159445773       0.00000000000000
C       -0.01011874753548      -3.38482395475182       0.00000000000000
N       -1.10587793745895      -2.52151055403438       0.00000000000000
H      -10.41170167076914      -0.11586582301191       0.00000000000000
C        2.43651358036049      -3.24389281365683       0.00000000000000
```

```
C        2.48327238950320     -4.62653095086246      0.00000000000000
C        1.28697975285501     -5.43955724180865      0.00000000000000
C        0.01704368746808     -4.77349612328418      0.00000000000000
H       10.41170167076914      0.11586582301191      0.00000000000000
H        3.44670109349714     -5.13513506649372      0.00000000000000
H       -0.90212820819461     -5.35907918165227      0.00000000000000
C       -1.37223304039898      6.84202845382319      0.00000000000000
H       -2.33662952054916      7.34435293435418      0.00000000000000
H       -0.47793957454837      7.46129150188085      0.00000000000000
H      -10.19687917500584     -1.96541785565724      0.00000000000000
C        1.37223304039898     -6.84202845382319      0.00000000000000
H        2.33662952054916     -7.34435293435418      0.00000000000000
H        0.47793957454837     -7.46129150188085      0.00000000000000
C       -2.88251187554103     -0.14846924343272      0.00000000000000
C        2.88251187554103      0.14846924343272      0.00000000000000
C       -4.09912099909605     -0.31880678502605      0.00000000000000
C        4.09912099909605      0.31880678502605      0.00000000000000
C        9.74556866304419      0.97573343713644      0.00000000000000
H        8.39080294802441     -1.36934345218959      0.00000000000000
H        5.94417425381302     -1.64801715980732      0.00000000000000
H        7.88952550799807      2.94800701579591      0.00000000000000
H       -5.94417425381302      1.64801715980732      0.00000000000000
C        5.49988945467395      0.48217033323473      0.00000000000000
C        6.09359558670109      1.78333929935476      0.00000000000000
C        7.46371046201515      1.94457150961328      0.00000000000000
C        8.35853272640897      0.81457589537811      0.00000000000000
C        7.74616496624533     -0.49035301891319      0.00000000000000
C        6.37575183350633     -0.64866002661434      0.00000000000000
H        5.44454110252079      2.65780360291638      0.00000000000000
C       -5.49988945467395     -0.48217033323473      0.00000000000000
C       -6.09359558670109     -1.78333929935476      0.00000000000000
C       -7.46371046201515     -1.94457150961328      0.00000000000000
C       -8.35853272640897     -0.81457589537811      0.00000000000000
C       -7.74616496624533      0.49035301891319      0.00000000000000
C       -6.37575183350633      0.64866002661434      0.00000000000000
H       -5.44454110252079     -2.65780360291638      0.00000000000000
H       -7.88952550799807     -2.94800701579591      0.00000000000000
C       -9.74556866304419     -0.97573343713644      0.00000000000000
H       -8.39080294802441      1.36934345218959      0.00000000000000
```

The triplet-optimized D_{2h} geometry of molecule $\boldsymbol{L}$ is given below (BLYP/TZP level of theory).
Total bonding energy: -11.131136314102742 $a.u.$

```
44
C28H12N4 optimized with ADF in AMS.
C       -0.00000000000000     -1.22411609603295     -6.20526211023079
H       -0.00000000000000     -2.16345066267519     -1.23914798051841
H       -0.00000000000000     -2.16345066267519      1.23914798051841
H        0.00000000000000      2.16576682307618     -5.66012211604852
N        0.00000000000000      2.21992819032290     11.09634238873075
C       -0.00000000000000     -1.20979170385599    -10.50378936008663
H       -0.00000000000000      2.17039922582181     -8.12700691073829
H       -0.00000000000000     -2.16576682307618     -5.66012211604852
C       -0.00000000000000      0.00000000000000     -9.76296625858532
H        0.00000000000000     -2.17039922582181      8.12700691073829
C        0.00000000000000     -1.22165404693183     -0.69415557488604
N       -0.00000000000000     -2.21992819032290    -11.09634238873075
C        0.00000000000000      1.20979170385599    -10.50378936008663
```

```
C        0.00000000000000      -1.22165404693183       0.69415557488604
C        0.00000000000000       0.00000000000000       1.42142345517762
C       -0.00000000000000       1.22165404693183       0.69415557488604
C       -0.00000000000000       1.22165404693183      -0.69415557488604
C       -0.00000000000000       0.00000000000000      -1.42142345517762
C       -0.00000000000000      -1.22490549287753      -7.58780098087883
H        0.00000000000000       2.16576682307618       5.66012211604852
H        0.00000000000000       2.16345066267519       1.23914798051841
C        0.00000000000000       0.00000000000000       5.47348452059953
H        0.00000000000000       2.16345066267519      -1.23914798051841
C       -0.00000000000000      -1.22411609603295       6.20526211023079
C       -0.00000000000000      -1.22490549287753       7.58780098087883
C        0.00000000000000       0.00000000000000       8.32234260741991
C        0.00000000000000       1.22490549287753       7.58780098087883
C        0.00000000000000       1.22411609603295       6.20526211023079
H        0.00000000000000      -2.17039922582181      -8.12700691073829
H       -0.00000000000000      -2.16576682307618       5.66012211604852
C       -0.00000000000000       0.00000000000000      -5.47348452059953
C       -0.00000000000000      -1.20979170385599      10.50378936008663
N        0.00000000000000       2.21992819032290     -11.09634238873075
N       -0.00000000000000      -2.21992819032290      11.09634238873075
C        0.00000000000000       1.20979170385599      10.50378936008663
C        0.00000000000000       1.22411609603295      -6.20526211023079
C        0.00000000000000       1.22490549287753      -7.58780098087883
C       -0.00000000000000       0.00000000000000      -8.32234260741991
C       -0.00000000000000       0.00000000000000      -2.83729529357031
C        0.00000000000000       0.00000000000000       2.83729529357031
C       -0.00000000000000       0.00000000000000      -4.06240189277498
C        0.00000000000000       0.00000000000000       4.06240189277498
C        0.00000000000000       0.00000000000000       9.76296625858532
H       -0.00000000000000       2.17039922582181       8.12700691073829
```

The triplet-optimized C_{2h} geometry of molecule $\boldsymbol{S}$ is given below (BLYP/TZP level of theory).
Total bonding energy: -10.315426748220721 $a.u.$

```
40
C24H10N6 optimized with ADF in AMS.
C       -3.29682143948277       0.72118054188573       0.00000000000000
C       -2.75584592773433      -0.60918306904103       0.00000000000000
N       -2.23836800106932       1.62554665586360       0.00000000000000
C       -4.66474222541586       0.96461981587275       0.00000000000000
C       -3.64359015512497      -1.70771432844440       0.00000000000000
C       -5.01083319380143      -1.48351697643797       0.00000000000000
C       -5.54702647201785      -0.15171330629033       0.00000000000000
N        8.62871969719202       1.93946075704300       0.00000000000000
H       -5.06195187062772       1.97802061323854       0.00000000000000
H        3.26236877299436       2.72776359127281       0.00000000000000
C       -1.31831417184317      -0.48394518469552       0.00000000000000
H       -3.26236877299436      -2.72776359127281       0.00000000000000
H       -5.69840925491024      -2.32611149815264       0.00000000000000
C       -1.02509955197532       0.93121990509070       0.00000000000000
C        0.27556012459595       1.43040909075843       0.00000000000000
C        1.31831417184317       0.48394518469552       0.00000000000000
C        1.02509955197532      -0.93121990509070       0.00000000000000
C       -0.27556012459595      -1.43040909075843       0.00000000000000
H       -0.47955787050839      -2.49987473509340       0.00000000000000
H        0.47955787050839       2.49987473509340       0.00000000000000
C        2.75584592773433       0.60918306904103       0.00000000000000
```

C	3.29682143948277	-0.72118054188573	0.00000000000000
N	2.23836800106932	-1.62554665586360	0.00000000000000
N	7.96449308570473	-2.44954603444705	0.00000000000000
C	3.64359015512497	1.70771432844440	0.00000000000000
C	5.01083319380143	1.48351697643797	0.00000000000000
C	5.54702647201785	0.15171330629033	0.00000000000000
C	4.66474222541586	-0.96461981587275	0.00000000000000
N	-7.96449308570473	2.44954603444705	0.00000000000000
H	5.69840925491024	2.32611149815264	0.00000000000000
H	5.06195187062772	-1.97802061323854	0.00000000000000
C	-6.97894554984883	0.05547587967166	0.00000000000000
C	-7.88891616805757	-1.03108486658144	0.00000000000000
C	-7.53458292218716	1.35977542883174	0.00000000000000
N	-8.62871969719202	-1.93946075704300	0.00000000000000
C	6.97894554984883	-0.05547587967166	0.00000000000000
C	7.88891616805757	1.03108486658144	0.00000000000000
C	7.53458292218716	-1.35977542883174	0.00000000000000
H	-2.33397447152207	2.63426412183599	0.00000000000000
H	2.33397447152207	-2.63426412183599	0.00000000000000

References

(1) te Velde, G.; Bickelhaupt, F. M.; Baerends, E. J.; Fonseca Guerra, C.; van Gisbergen, S. J. A.; Snijders, J. G.; Ziegler, T. Chemistry with ADF. *J. Comput. Chem.* **2001**, *22*, 931–967.

(2) Becke, A. D. Density-functional exchange-energy approximation with correct asymptotic behavior. *Phys. Rev. A* **1988**, *38*, 3098–3100.

(3) Lee, C.; Yang, W.; Parr, R. G. Development of the Colle-Salvetti correlation-energy formula into a functional of the electron density. *Phys. Rev. B* **1988**, *37*, 785–789.

(4) Van Lenthe, E.; Baerends, E. J. Optimized Slater-type basis sets for the elements 1–118. *J. Comput. Chem.* **2003**, *24*, 1142–1156.

(5) Becke, A. D. A multicenter numerical integration scheme for polyatomic molecules. *J. Chem. Phys.* **1988**, *88*, 2547–2553.

(6) Franchini, M.; Philipsen, P. H. T.; Visscher, L. The Becke Fuzzy Cells Integration Scheme in the Amsterdam Density Functional Program Suite. *J. Comput. Chem.* **2013**, *34*, 1819–1827.

(7) Jacobsen, H.; Bérces, A.; Swerhone, D. P.; Ziegler, T. Analytic second derivatives of molecular energies: a density functional implementation. *Comput. Phys. Commun.* **1997**, *100*, 263–276.

(8) Ruedenberg, K.; Sundberg, K. R. In *"Quantum Science: Methods and Structure. A Tribute to Per-Olov Löwdin"*; Calais, J.-L., Goscinski, O., Linderberg, J., Öhrn, Y., Eds.; Springer US: Boston, MA, 1976; pp 505–515.

(9) Cheung, L. M.; Sundberg, K. R.; Ruedenberg, K. Dimerization of carbene to ethylene. *J. Am. Chem. Soc.* **1978**, *100*, 8024–8025.

(10) Cheung, L. M.; Sundberg, K. R.; Ruedenberg, K. Electronic rearrangements during chemical reactions. II. Planar dissociation of ethylene. *Int. J. Quantum Chem.* **1979**, *16*, 1103–1139.

(11) Roos, B. O.; Taylor, P. R.; Sigbahn, P. E. A complete active space SCF method (CASSCF) using a density matrix formulated super-CI approach. *Chem. Phys.* **1980**, *48*, 157–173.

(12) Johnson, R. P.; Schmidt, M. W. The sudden polarization effect: MC-SCF calculations on planar and 90.degree. twisted methylenecyclopropene. *J. Am. Chem. Soc.* **1981**, *103*, 3244–3249.

(13) Feller, D. F.; Schmidt, M. W.; Ruedenberg, K. Concerted dihydrogen exchange between ethane and ethylene. SCF and FORS calculations of the barrier. *J. Am. Chem. Soc.* **1982**, *104*, 960–967.

(14) Ruedenberg, K.; Schmidt, M. W.; Gilbert, M. M.; Elbert, S. Are atoms intrinsic to molecular electronic wavefunctions? I. The FORS model. *Chem. Phys.* **1982**, *71*, 41–49.

(15) Shepard, R. *Advances in Chemical Physics*; John Wiley & Sons, Ltd, 1987; pp 63–200.

(16) Barca, G. M. J. *et al.* Recent developments in the general atomic and molecular electronic structure system. *J. Chem. Phys.* **2020**, *152*, 154102.

(17) Woon, D. E.; Dunning, J., Thom H. Gaussian basis sets for use in correlated molecular calculations. V. Core-valence basis sets for boron through neon. *J. Chem. Phys.* **1995**, *103*, 4572–4585.

(18) Zahariev, F. *et al.* The General Atomic and Molecular Electronic Structure System (GAMESS): Novel Methods on Novel Architectures. *J. Chem. Theory Comput.* **2023**, *19*, 7031–7055.
(19) Hirao, K. Multireference Møller—Plesset method. *Chem. Phys. Lett.* **1992**, *190*, 374–380.
(20) Hirao, K. Multireference Møller—Plesset perturbation theory for high-spin open-shell systems. *Chem. Phys. Lett.* **1992**, *196*, 397–403.
(21) Hirao, K. Multireference Møller–Plesset perturbation treatment of potential energy curve of N2. *Int. J. Quantum Chem.* **1992**, *44*, 517–526.
(22) Hirao, K. State-specific multireference Møller—Plesset perturbation treatment for singlet and triplet excited states, ionized states and electron attached states of H2O. *Chem. Phys. Lett.* **1993**, *201*, 59–66.
(23) Nakano, H.; Nakayama, K.; Hirao, K.; Dupuis, M. Transition state barrier height for the reaction H2CO→H2+CO studied by multireference Mo/ller–Plesset perturbation theory. *J. Chem. Phys.* **1997**, *106*, 4912–4917.
(24) Hashimoto, T.; Nakano, H.; Hirao, K. Theoretical study of valence and Rydberg excited states of benzene revisited. *J. Mol. Struct.* **1998**, *451*, 25–33.
(25) Lowdin, P.-O. Present Situation of Quantum Chemistry. *J. Phys. Chem.* **1957**, *61*, 55–68.
(26) Pipek, J.; Mezey, P. G. A fast intrinsic localization procedure applicable for abinitio and semiempirical linear combination of atomic orbital wave functions. *J. Chem. Phys.* **1989**, *90*, 4916–4926.
(27) Pulay, P.; Hamilton, T. P. UHF natural orbitals for defining and starting MC-SCF calculations. *J. Chem. Phys.* **1988**, *88*, 4926–4933.
(28) Heisenberg, W. Zur Theorie des Ferromagnetismus. *Z. Phys.* **1928**, *49*, 619–636.
(29) Dirac, P. A. M.; Polkinghorne, J. C. The Principles of Quantum Mechanics. *Phys. Today* **1958**, *11*, 32–33.
(30) Van Vleck, J. *The Theory of Electric and Magnetic Susceptibilities*; Internat. Ser. Monogr. Phys.; Clarendon Press, 1932.
(31) de P. R. Moreira, I.; Illas, F. Ab initio theoretical comparative study of magnetic coupling in $KNiF_3$sand K_2 NiF_4s. *Phys. Rev. B* **1997**, *55*, 4129–4137.
(32) Durand, P.; Malrieu, J.-P. *Advances in Chemical Physics*; John Wiley & Sons, Ltd, 1987; pp 321–412.
(33) Schmidt, E. Zur Theorie der linearen und nichtlinearen Integralgleichungen. *Math. Ann.* **1907**, *63*, 433–476.
(34) Bloch, C. Sur la théorie des perturbations des états liés. *Nucl. Phys.* **1958**, *6*, 329–347.
(35) des Cloizeaux, J. Extension d'une formule de Lagrange à des problèmes de valeurs propres. *Nucl. Phys.* **1960**, *20*, 321–346.
(36) Reta, D.; Moreira, I. d. P. R.; Illas, F. Magnetic Coupling Constants in Three Electrons Three Centers Problems from Effective Hamiltonian Theory and Validation of Broken Symmetry-Based Approaches. *J. Chem. Theory Comput.* **2016**, *12*, 3228–3235.
(37) Ovchinnikov, A. A. Multiplicity of the ground state of large alternant organic molecules with conjugated bonds. *Theor. Chim. Acta* **1978**, *47*, 297–304.
(38) Perdew, J. P.; Burke, K.; Ernzerhof, M. Generalized Gradient Approximation Made Simple. *Phys. Rev. Lett.* **1996**, *77*, 3865–3868.
(39) Perdew, J. P.; Burke, K.; Ernzerhof, M. Generalized Gradient Approximation Made Simple [Phys. Rev. Lett. 77, 3865 (1996)]. *Phys. Rev. Lett.* **1997**, *78*, 1396–1396.
(40) Geuenich, D.; Hess, K.; Köhler, F.; Herges, R. Anisotropy of the Induced Current Density (ACID), a General Method To Quantify and Visualize Electronic Delocalization. *Chem. Rev.* **2005**, *105*, 3758–3772.
(41) Adamo, C.; Barone, V. Toward reliable density functional methods without adjustable parameters: The PBE0 model. *J. Chem. Phys.* **1999**, *110*, 6158–6170.
(42) Ernzerhof, M.; Scuseria, G. E. Assessment of the Perdew–Burke–Ernzerhof exchange-correlation functional. *J. Chem. Phys.* **1999**, *110*, 5029–5036.
(43) Clar, E. *The aromatic sextet*; New York, Wiley, 1972.
(44) Bultinck, P.; Ponec, R.; Van Damme, S. Multicenter bond indices as a new measure of aromaticity in polycyclic aromatic hydrocarbons. *J. Phys. Org. Chem.* **2005**, *18*, 706–718.
(45) Feixas, F.; Matito, E.; Poater, J.; Solà, M. Quantifying aromaticity with electron delocalisation measure. *Chem. Soc. Rev.* **2015**, *44*, 6434–6451.
