# Supplementary material for: Pathway to Polyradicals: A Planar and Fully π-Conjugated Organic Tetraradical(oid)
Source: J Phys Chem Lett. 2024 May 8;15(19):5243–9. doi: 10.1021/acs.jpclett.4c00686 (PMC11103692; doi:10.1021/acs.jpclett.4c00686)
Supplement: Supplementary file 2 — jz4c00686_si_002.pdf [file jz4c00686_si_002.pdf]

jz-2024-00686w.R1

Name: Peer Review Information for "Pathway to Polyradicals: A Planar and Fully π-Conjugated Organic Tetraradical(oid)"

First Round of Reviewer Comments

Reviewer: 1

Comments to the Author

In this study, the authors present a proposed method aimed at stabilizing the ground state of polyradical(oid)s while simultaneously enabling the thermal accessibility of higher spin states. The approach involves the amalgamation of two planar, fully π-conjugated diradical(oid)s to yield a planar and cross-conjugated tetraradical(oid). Employing multireference quantum chemistry methods, the authors demonstrate that the designed tetraradical(oid) achieves stabilization through aromaticity and delocalization within the π-system, resulting in the existence of six thermally accessible spin states within a narrow energy range of ~2 kcal/mol. A detailed analysis of the electronic structure of these six states reveals a frontier π-system comprising two weakly interacting subsystems: aromatic cycles and four unpaired electrons. The conjugation between unpaired electrons, which typically favors closed-shell structures, is offset by the delocalization and aromaticity of the bridging groups, facilitating a synergistic cross-coupling between the two diradical(oid) subunits to stabilize the tetraradical(oid) electronic structure. While the overall description is reasonable, the experimental support such as SQUID or ESR for this molecule is highly recommendable, though this type of measurement may require a strong collaboration with the experimentalists in the related area.

Reviewer: 2

Comments to the Author

In the manuscript Bofill and co-workers outline a new approach to design high-spin polyradicaloids and explore one tetraradicaloid species computationally at CASSCF level (and with KS DFT). It is based on the cross-conjugation between two moieties that individually can adapt diradicaloid character. In principle I think the study is highly interesting and it provides a new direction that likely also can be addressed experimentally. Still, I also have criticism that should be addressed.

In the computational part of the manuscript the authors explore just one compound (named LS) and its two separate components (L and S). Both of the separate components have dicyanomethylene moieties as radical centers. If there instead would be two regular methylene moieties at either the L or S unit I expect that the diradicaloid state would be the lowest, not any tetraradicaloid state. This should be tested by the authors.

The aromaticity analysis is only based on an ACID with the PBE0 functional. I had expected a more comprehensive analysis here. The authors conclude early in the manuscript that KS DFT is not suitable, but for the aromaticity analysis they use exactly KS DFT. Why? How much does the "global aromaticity" vary with functional used? Especially what does CAM-B3LYP tell? I would also like to see something more than just an ACID plot, e.g., results with an electronic aromaticity index. Those results can be placed in the Supporting Information but they must be available to the skeptical reader.

Finally, the authors label the approach as "a general strategy" in the Abstract. However, they only give one example (not very general). They must explain how their design approach can be generalized. As the generality relies on the fact that the central ring system attains a global aromatic character, I believe there is a limit in size which most likely is not much larger than the LS species they now explore.

Author's Response to Peer Review Comments:

Senior Editor
The Journal of Physical Chemistry Letters

April 24, 2024

Manuscript ID: jz-2024-00686w
Manuscript title: **"Pathway to Polyradicals: A Planar and Fully π-Conjugated Organic Tetraradical(oid)."**

Authors: Sergi Betkhoshvili, Ibério de P.R. Moreira, Jordi Poater and Josep Maria Bofill

Dear Editor,

We have carefully examined the report of the reviewers and concluded that, despite some well-grounded criticisms, the overall judgment is essentially favorable. Therefore, we have prepared a new, improved version of the manuscript that considers the essential points and includes a detailed reply to all referees' remarks.

We are also submitting the main text of the manuscript with tracked changes (as "Supporting Information for Review Only"), by the following color code: added text is wavy-underlined and blue, ~~and discarded text is struck out and red~~. It is important to consider that the two reviewers emphasize the importance of this study based on the analysis of organic tetraradical(oid) species formed by aromatic cycles and four unpaired electrons.

Yours sincerely,

Prof. Josep Maria Bofill

Reviewer 1

Answers and revisions made in the manuscript according to the comments and the recommended changes of the first Reviewer

In this study, the authors present a proposed method aimed at stabilizing the ground state of polyradical(oid)s while simultaneously enabling the thermal accessibility of higher spin states. The approach involves the amalgamation of two planar, fully π-conjugated diradical(oid)s to yield a planar and cross-conjugated tetraradical(oid). Employing multireference quantum chemistry methods, the authors demonstrate that the designed tetraradical(oid) achieves stabilization through aromaticity and delocalization within the π-system, resulting in the existence of six thermally accessible spin states within a narrow energy range of ~2 kcal/mol. A detailed analysis of the electronic structure of these six states reveals a frontier π-system comprising two weakly interacting subsystems: aromatic cycles and four unpaired electrons. The conjugation between unpaired electrons, which typically favors closed-shell structures, is offset by the delocalization and aromaticity of the bridging groups, facilitating a synergistic cross-coupling between the two diradical(oid) subunits to stabilize the tetraradical(oid) electronic structure. While the overall description is reasonable, the experimental support such as SQUID or ESR for this molecule is highly recommendable, though this type of measurement may require a strong collaboration with the experimentalists in the related area.

We thank reviewer 1 for the positive evaluation of our manuscript. Furthermore, we fully agree with the necessity of experimental methods such as SQUID and ESR to verify these results. Indeed, this is what we will elaborate on when we find experimental collaborators to synthesize this molecule and make magnetic measurements on it. Nevertheless, we must mention that the strategy used to build the proposed tetraradical can be extended to design polyradicals of any size, including other tetraradicals

with different properties. Currently, we have preliminary results on other polyradicals that fully exemplify the strategy we proposed in this Letter. For example, we have just found a new tetraradical that is topologically equivalent to the proposed tetraradical and has an even higher tetraradical character with thermally accessible spin states within a narrower energy range of 0.32 kcal/mol. Therefore, the strategy to design polyradicals has been repeatedly and consistently shown to work for polyradicals that we are currently exploring. These guidelines will serve experimentalists to design polyradicals with desired properties.

Reviewer 2

Answers and revisions made in the manuscript according to the comments and the recommended changes of the second Reviewer

In the manuscript Bofill and co-workers outline a new approach to design high-spin polyradicaloids and explore one tetraradicaloid species computationally at CASSCF level (and with KS DFT). It is based on the cross-conjugation between two moieties that individually can adapt diradicaloid character. In principle I think the study is highly interesting and it provides a new direction that likely also can be addressed experimentally. Still, I also have criticism that should be addressed.

We thank reviewer 2 for the overall positive evaluation of our manuscript. We have considered the criticism of reviewer 2 and provided detailed answers to their inquiries.

In the computational part of the manuscript the authors explore just one compound (named LS) and its two separate components (L and S). Both of the separate components have dicyanomethylene moieties as radical centers. If there instead would be two regular methylene moieties at either the L or S unit I expect that the diradicaloid state would be the lowest, not any tetraradicaloid state. This should be tested by the authors.

In response to reviewer 2, we acknowledge that the reasoning for expecting diradical(oid) ground state for hydrogen-substituted ***LS*** (all cyano groups are substituted with hydrogen; this compound is further referred to as ***LSH***) is legitimate, as cyano groups stabilize radical centers, by π-conjugation. However, the effect of cyano groups alone would be insufficient to bring about tetraradical electronic structure. Both ***LS*** and ***LSH*** have the same topology of π-conjugation and the same source of aromatic stabilization. Comparing results from CASSCF(4,4) and CASSCF(16,16) for these molecules, we find that ***LSH*** has almost equal tetraradical and diradical characters as ***LS*** based on the occupation numbers of frontier Natural Orbitals (NOs). CASSCF results for ***LS*** and ***LSH*** are shown in **Tables R1** to **R4** below. These results unequivocally show that the electronic structure of ***LSH*** shares remarkable similarity with our proposed tetraradical ***LS*** since occupation numbers of frontier NOs are close for each state. As long as each diradical subunit ***L*** and ***S*** does not acquire significant electric dipole moment (i.e., there is no significant charge transfer between radical centers), ***LS*** and ***LSH*** correspond to the extreme cases. Any derivative of ***LS***, which includes hydrogen-substituted subunit ***L*** or ***S***, would show similar and intermediate cases between ***LS*** and ***LSH***, in terms of the energy range of the spin spectrum and polyradical character indices. Hence, we verify that the source of stabilization of a tetraradical electronic structure in these compounds mainly comes from the topology of π-conjugation and aromaticity of bridging groups between radical centers.

These results for ***LSH*** have been included in Section S6 of the Supporting Information of the manuscript.

Table R1. CASSCF(16,16) results for ***LSH*** from UHF Quintet natural orbitals. cc-pVDZ basis set. C_{2h} optimized geometry from ADF/BLYP/TZP/Non-relativistic.

State	Symmetry	Energy (a.u.)	CASSCF natural orbitals symmetry, identity and occupation number												ΔE from ground state (cm^{-1})
			HONO − 1			HONO			LUNO			LUNO + 1			
S_0	A_g	-1561.9113563163	A_u	S_+	1.263	A_u	L_+	1.049	B_g	L_-	0.948	B_g	S_-	0.742	0.00
T_0	B_u	-1561.9108982411	A_u	S_+	1.264	B_g	L_-	1.001	A_u	L_+	0.997	B_g	S_-	0.740	100.54
S_1	A_g	-1561.9074478913	A_u	C_0	1.081	B_g	C_1	1.010	B_g	C_1'	0.976	A_u	C_2	0.936	857.80
T_1	B_u	-1561.9074101414	A_u	D_0^*	1.074	A_u	D_2^*	0.997	B_g	S_-	0.994	B_g	L_-	0.937	866.09
T_2	A_g	-1561.9073234317	A_u	C_0	1.060	B_g	C_1	1.026	B_g	C_1'	0.971	A_u	C_2	0.945	885.12
Q_0	A_g	-1561.9068303423	A_u	C_0	1.005	A_u	C_2	1.000	B_g	D_1	1.000	B_g	D_1'	0.945	993.34

* D means distorted orbitals coming from same identities as C orbitals.

Table R2. CASSCF(4,4) results for ***LSH*** from UHF Quintet natural orbitals. cc-pVDZ basis set. C_{2h} optimized geometry from ADF/BLYP/TZP/Non-relativistic.

State	Symmetry	Energy (a.u.)	CASSCF natural orbitals symmetry, identity and occupation number												ΔE from ground state (cm^{-1})
			HONO − 1			HONO			LUNO			LUNO + 1			
S_0	A_g	-1561.7656766156	A_u	S_+	1.142	A_u	L_+	1.036	B_g	L_-	0.962	B_g	S_-	0.860	0.00
T_0	B_u	-1561.7655429785	A_u	S_+	1.142	B_g	L_-	1.001	A_u	L_+	0.998	B_g	S_-	0.860	29.33
S_1	A_g	-1561.7644852268	A_u	C_0	1.049	B_g	C_1	1.006	B_g	C_1'	0.987	A_u	C_2	0.959	261.48
T_1	B_u	-1561.7644370946	A_u	L_+	1.045	B_g	S_-	0.998	A_u	S_+	0.997	B_g	L_-	0.959	272.04
T_2	A_g	-1561.7644279814	A_u	C_0	1.034	B_g	C_1	1.017	B_g	C_1'	0.983	A_u	C_2	0.860	274.04
Q_0	A_g	-1561.7642517325	A_u	D_0^*	1.000	B_g	D_1	1.000	A_u	D_2	1.000	B_g	D_1'	1.000	312.73

* D means distorted orbitals coming from same identities as C orbitals.

Table R3. CASSCF(16,16) results for ***LS*** from UHF Quintet natural orbitals. cc-pVDZ basis set. C_{2h} optimized geometry from ADF/BLYP/TZP/Non-relativistic.

State	Symmetry	Energy (a.u.)	CASSCF natural orbitals symmetry, identity and occupation number												ΔE from ground state (cm^{-1})
			HONO − 1			HONO			LUNO			LUNO + 1			
S_0	A_g	-2295.7831909607	A_u	S_+	1.221	A_u	L_+	1.043	B_g	L_-	0.955	B_g	S_-	0.781	0.00
T_0	B_u	-2295.7829323238	A_u	S_+	1.222	B_g	L_-	1.000	A_u	L_+	0.998	B_g	S_-	0.780	56.76
T_1	B_u	-2295.7807627335	A_u	L_+	1.058	A_u	S_+	0.997	B_g	S_-	0.995	B_g	L_-	0.948	532.93
S_1	A_g	-2295.7807543493	A_u	C_0	1.059	B_g	C_1	1.011	B_g	C_1'	0.977	A_u	C_2	0.951	534.77
T_2	A_g	-2295.7806889597	A_u	C_0	1.043	B_g	C_1	1.021	B_g	C_1'	0.975	A_u	C_2	0.959	549.13
Q_0	A_g	-2295.7804446812	A_u	C_0	1.002	A_u	C_2	0.999	B_g	L_-	0.999	B_g	S_-	0.997	602.74

Table R4. CASSCF(4,4) results for ***LS*** from UHF Quintet natural orbitals. cc-pVDZ basis set. C_{2h} optimized geometry from ADF/BLYP/TZP/Non-relativistic.

State	Symmetry	Energy (a.u.)	CASSCF natural orbitals symmetry, identity and occupation number												ΔE from ground state (cm^{-1})
			HONO − 1			HONO			LUNO			LUNO + 1			
S_0	A_g	-2295.6577083553	A_u	S_+	1.155	A_u	L_+	1.038	B_g	L_-	0.961	B_g	S_-	0.846	0.00
T_0	B_u	-2295.6575974661	A_u	S_+	1.155	B_g	L_-	1.001	A_u	L_+	0.998	B_g	S_-	0.846	24.34
S_1	A_g	-2295.6565732806	A_u	C_0	1.046	B_g	C_1	1.011	B_g	C_1'	0.982	A_u	C_2	0.961	249.12
T_1	B_u	-2295.6565606467	A_u	L_+	1.046	B_g	S_-	0.999	A_u	S_+	0.998	B_g	L_-	0.957	251.89
T_2	A_g	-2295.6565364226	A_u	C_0	1.033	B_g	C_1	1.019	B_g	C_1'	0.981	A_u	C_2	0.967	257.21
Q_0	A_g	-2295.6564170504	B_g	L_-	1.000	A_u	C_0	1.000	A_u	C_2	1.000	B_g	S_-	1.000	283.41

The aromaticity analysis is only based on an ACID with the PBE0 functional. I had expected a more comprehensive analysis here. The authors conclude early in the manuscript that KS DFT is not suitable, but for the aromaticity analysis they use exactly KS DFT. Why? How much does the "global aromaticity" vary with functional used? Especially what does CAM-B3LYP tell? I would also like to see something more than just an ACID plot, e.g., results with an electronic aromaticity index. Those results can be placed in the Supporting Information but they must be available to the skeptical reader.

As suggested by the reviewer, in Section S4 of the Supporting Information, we included a benchmark of PBE-X functional with variable % of Hartree-Fock exchange to explore charge and spin localization due to electron correlation effects in DFT. The purpose of this benchmark has been twofold: 1. To analyze how the description of different spin states can be approximated by inherently insufficient monoconfigurational methods for the full description of the multiconfigurational system; and 2. To find the appropriate exchange-correlation functional, which gives qualitatively correct results for some of the spin states. It had been mentioned that broken-symmetry unrestricted Hartree-Fock singlet described the tetraradical electronic structure of the ground state qualitatively correctly. However, HF or KS-DFT are inherently insufficient to capture all spin states of the multiconfigurational tetraradical. Hence, when we apply monoconfigurational methods to these systems, we must always verify whether they describe the qualitative electronic structure of target states properly. This benchmark revealed that a significant number of states cannot be described properly by PBE-X (X means variability of % of Hartree-Fock exchange), or by any exchange-correlation functional, because monoconfigurational Hamiltonian lacks higher roots that multiconfigurational Hamiltonian includes. Hence, we cannot rely on KS-DFT alone to study this molecule. However, if some exchange-correlation functional properly describes the qualitative electronic structure of some spin state of the molecule, we can use this monoconfigurational approximation for the demonstration of some of the properties such as aromaticity as shown by Anisotripy of Induced Current Density (ACID). It is important to stress that the property we are demonstrating by ACID is the aromaticity of the molecule, which is already plausible from resonance structures. It is important to remark that the aromaticity of the molecule can be inferred from CASSCF and CASCI calculations as shown in the Supporting Information. Since we verified that PBE0 captures the qualitative features of the electronic structure correctly for singlet and quintet states, we used it to show the aromaticity of the molecule, which is persistent across different spin states.

Moreover, and as suggested by the reviewer, we have also computed the Mulicenter Index (MCI) of aromaticity for every ring in order to further demonstrate the aromaticity of the proposed molecule. It is also important to stress that MCI values are hardly affected by the change of exchange-correlation functional, even by using CAM-B3LYP. These results are given in **Table R5** and ring numbering is given in **Figure R1**.

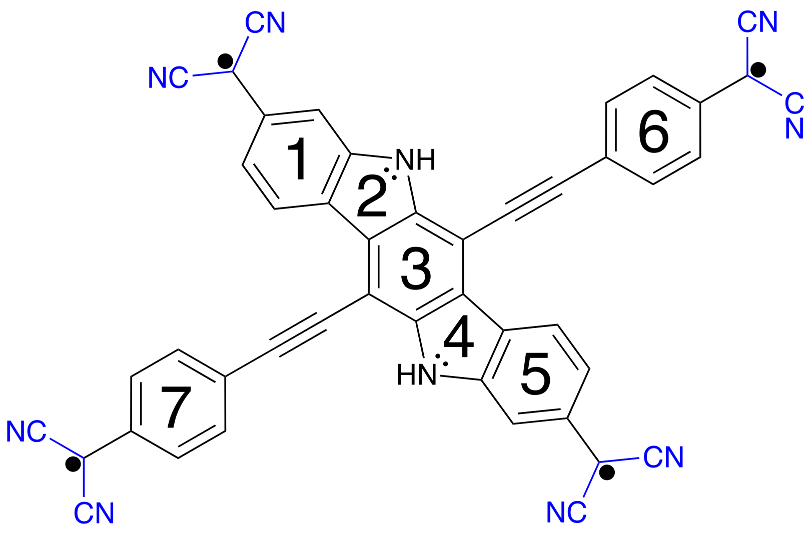

Figure R1. Ring numbering of ***LS***.

Table R5. Multicenter Index (MCI) results for Singlet (*S*), Triplet (*T*), and Quintet (*Q*) tetraradical(oid) spin states of ***LS*** with different exchange-correlation functionals. Rings (R1, R2 ...) are numbered according to **Figure R1**.

Functional	State	Multicenter Index						
		R1	R2	R3	R4	R5	R6	R7
BLYP	S	0.028	0.014	0.014	0.014	0.028	0.036	0.036
	T	0.030	0.015	0.014	0.014	0.029	0.033	0.035
	Q	0.031	0.013	0.017	0.013	0.031	0.038	0.038
B3LYP	S	0.030	0.012	0.017	0.012	0.030	0.038	0.038
	T	0.032	0.013	0.018	0.012	0.031	0.037	0.038
	Q	0.032	0.012	0.019	0.012	0.032	0.039	0.039
CAM-B3LYP	S	0.032	0.011	0.021	0.011	0.032	0.039	0.039
	T	0.034	0.011	0.021	0.011	0.032	0.038	0.039
	Q	0.033	0.011	0.023	0.011	0.033	0.040	0.040
PBE0	S	0.031	0.012	0.018	0.012	0.031	0.038	0.038
	T	0.032	0.012	0.018	0.012	0.031	0.037	0.038
	Q	0.032	0.012	0.020	0.012	0.032	0.039	0.039

These results will be added as Figure S9 and Table S10 in Section S5 of the Supporting Information.

Finally, the authors label the approach as "a general strategy" in the Abstract. However, they only give one example (not very general). They must explain how their design approach can be generalized. As the generality relies on the fact that the central ring system attains a global aromatic character, I believe there is a limit in size which most likely is not much larger than the LS species they now explore.

In response to this criticism, the reason we label our approach as "a general strategy" is that by using this approach, one can design polyradicals with any practical number of unpaired electrons. As described in the manuscript, one must satisfy the proper topology of π-conjugation and requirements of the aromatic stabilization per diradical(oid) subunit.

The reason we claim this strategy to be general is that by using this approach we have designed a multitude of polyradicals ranging from triradicals to higher than octadecaradicals, which we are currently exploring in greater detail. Even though we only propose a single molecule in this manuscript, it already captures the most important necessary and sufficient conditions to build a tetraradical, and we can extend and generalize this approach readily by following the equivalent or similar topology of π-conjugation and requirements on aromatic stabilization. We emphasize that the limit of this strategy of polyradical design is not by any means obvious, and one can conjecture that we can design polyradical of any practical size.

Nevertheless, we acknowledge that the reader might not immediately identify the generality of the approach and we have included a short but conclusive explanation in the main text (page 7 of the manuscript) as follows:

"Our general approach can be summarized in the following way: 1) If the polyradical is built from merging diradicals or other polyradicals, one must ensure that π-conjugation is maintained throughout the resulting structure. 2) Within a given polyradical, each diradical subunit must have an aromatic stabilization with at least two or (more favorably) three benzene rings. 3) Aromatic rings can be shared between different subunits, similarly as the central benzene ring is shared between ***L*** and ***S*** subunits within tetraradical(oid) ***LS***. 4) Upon the design of a polyradical, one can take advantage of cross-conjugation to restrict the lower bound of polyradical character, as we did to restrict the minimal polyradical character of tetraradical(oid) ***LS*** to 2."

We thank both reviewers for positive and constructive comments that helped us to improve the manuscript.

I hope you will find the new version of the manuscript suitable for publication in The Journal of Physical Chemistry Letters.

Yours Sincerely,

Prof. Josep Maria Bofill
